# Population coding for visual and auditory quantity in human numerotopic maps

**DOI:** 10.1038/s42003-026-09752-2

**Published:** 2026-02-27

**Authors:** Garam Jeong, Joram Soch, Robert Trampel, Andreas Nieder, Michael A. Skeide

**Affiliations:** 1 Research Group Learning in Early Childhood, Max Planck Institute for Human Cognitive and Brain Sciences, Leipzig, Germany; 2 Institute for Psychology, Otto-von-Guericke-Universität Magdeburg Universitätsplatz 2, Magdeburg, Germany; 3 Department of Neurophysics, Max Planck Institute for Human Cognitive and Brain Sciences, Leipzig, Germany; 4 Animal Physiology Unit, Institute of Neurobiology, Eberhard-Karls-Universität Tübingen, Tübingen, Germany; 5 Institute for Child and Adolescent Psychiatry, Christian-Albrechts-Universität zu Kiel, Kiel, Germany

**Keywords:** Cognitive neuroscience, Perception

## Abstract

Neural populations that are able to extract quantitative information from multiple sensory domains are essential for the survival of numerous species. How quantity is encoded across different senses is far from understood. Here, we identified an overarching coding scheme for visual and auditory numerosity using high-field functional magnetic resonance imaging at 7 Tesla in humans. Based on a neurobiologically plausible model informed by electrophysiological data, we discovered hemodynamic responses revealing logarithmic Gaussian tuning to numerosity in both domains. Responses were organized topographically, forming numerotopic maps. We found several visual maps scattered over the association cortices and anatomically distinct auditory maps in superior temporal and premotor cortices. The present data shed light on the multisensory foundations of numerical information processing in the human brain. These insights open avenues for future research exploring how different species detect quantity in different sensory modalities.

## Abstract (web summary)

Functional magnetic resonance imaging at 7 Tesla reveals an overarching coding scheme for visual and auditory numerosity in the human brain.

## Main

Electrophysiological recordings from single neurons in the parietal and medial temporal lobe of human neurosurgical patients uncovered populations that selectively respond to simultaneously displayed dots of a certain numerosity. Neuronal response profiles revealed peak activity for one preferred numerosity out of the numerosities one to five and a gradual decrease of activity the more the number of dots deviated from the preferred value^1,2^.

Invasive recording techniques achieve unparalleled spatial and temporal resolution but suffer from selection bias to neurological patients and sampling bias to a relatively small set of predefined regions of interest. High-field magnetic resonance imaging, however, despite measuring neuronal activity only indirectly, revealed whole-brain wide neural populations with visually and haptically elicited hemodynamic response profiles similar to the electrophysiological response profile of numerosity-tuned neurons^3–5^. It is currently unknown, however, whether this common coding scheme also governs other special senses, such as audition. Audition is a critical test case for numerosity coding since, unlike vision, it primarily draws on temporal rather than spatial numerical information and is thus not confounded by spatial frequency. This also means that auditory numerosity processing does not operate in a simultaneous, but in a sequential mode of stimulus presentation. Moreover, previous work could show that visually and haptically elicited hemodynamic responses form topographic maps in which neighboring numerosities are represented in adjacent patches of cortex to minimize signal delay and energy consumption and to facilitate parallelized detection of numerical and non-numerical features^3–5^. It remains to be explored whether auditory responses also follow this functional mapping principle.

Here, we set out to explore the existence of cross-modal visual and auditory population coding for quantity. To this end, we conducted experiments in which human adult participants either viewed dot arrays or listened to beeps. Visual dot arrays were presented simultaneously with varying positions but constant luminance. Beeps were presented sequentially with randomly varying pitch. The numerosities one to five appeared 384 times in each modality while recording high-field magnetic resonance imaging data at 7 Tesla. Following previous electrophysiological and magnetic resonance imaging work, we employed a single-subject design in which twelve individual data sets served as replication units and not as measurement units.

We consistently found topographically organized neural populations with logarithmic Gaussian tuning to numerosity across senses, presentation modes and stimulus features. While sharing coding schemes and topographic mapping principles, these populations generated anatomically dissociable responses to either visual or auditory numerosity.

## Results

### Hemodynamic responses display logarithmic Gaussian tuning to visual and auditory numerosity

**Fig. 1:**
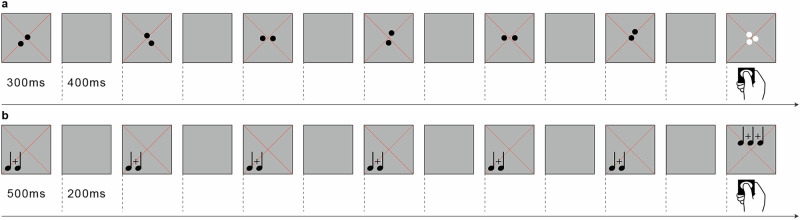
Stimuli and task. **a** In the visual experiment, participants viewed six different dot arrays per numerosity that were separately displayed for 300 ms and alternating with 400 ms of gray background (six trials = one block). Arrays appeared within a radius of 0.75° (visual angle) of a diagonal cross of thin red fixation lines. Summed surface area was kept constant and dot positions were randomly varied. Participants were instructed to press a button whenever white instead of black dots were shown. These catch trials occurred in 10% of all stimulus events. **b** In the auditory experiment, participants listened to sequences of beep tones played six times for 500 ms each, alternating with 200 ms of silence (= one block). Within each sequence, pitch was randomly varied between 333, 359, 392 and 440 kHz. Participants were instructed to press a button whenever 1000 kHz beeps (superscript notes) were played instead of 333 to 440 kHz beeps (subscript notes). These catch trials occurred in 10% of all stimulus events. **a**, **b** Numerosities one through five were first presented in ascending order, followed by a block of twenty items, then in descending order, followed by another block of twenty items before this cycle (five ascending blocks, block of twenty, five descending blocks, block of twenty) was repeated.

Participants were instructed to press a button whenever visual dot arrays were shown in different color or auditory beep sequences were presented in different pitch (Fig. 1). Across all participants and runs, the hit rate for these catch trials was 99.69% for the visual and 80.38% for the auditory experiments on average (Supplementary Table S1). This behavioral performance indicates that participants were paying attention to the stimuli.

**Fig. 2:**
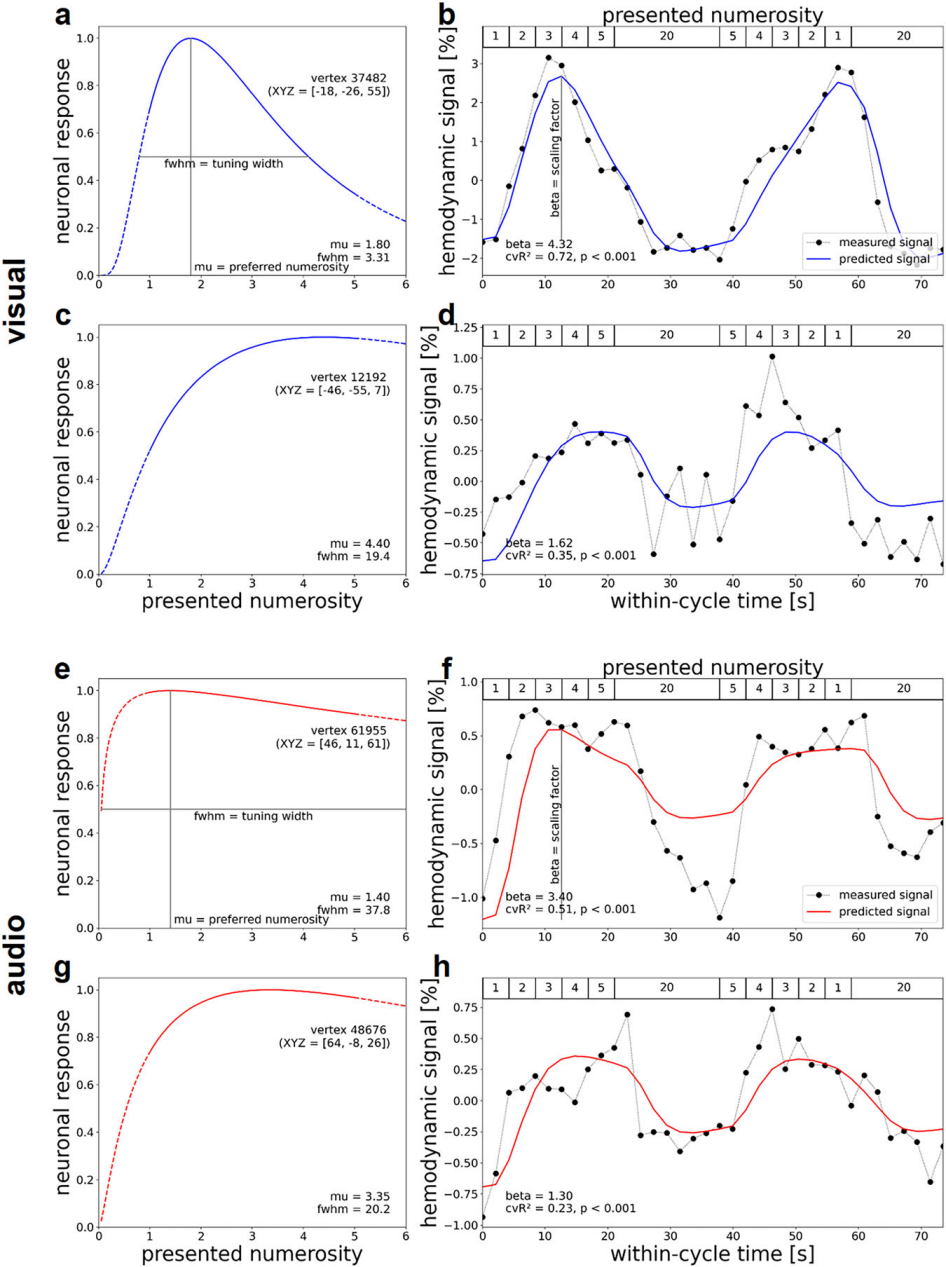
Neural numerosity tuning and hemodynamic activation time course. **a**–**h** Results of numerosity population receptive field analyses for an exemplary subject seeing visual stimuli (top, blue) and another subject hearing auditory stimuli (bottom, red). Rows **a**, **b** and **e**, **f** depict vertices preferring low-numerosity while rows **c**, **d** and **g**, **h** depict high-numerosity examples. **a**–**d** Responses in the left inferior parietal and occipital cortex were elicited by visual numerosities. **e**–**h** Responses in the left precentral gyrus and superior temporal cortex were elicited by auditory numerosities. **a**, **c**, **e**, **g** Logarithmic Gaussian tuning functions are shown for **a**, **e** a small-numerosity vertex and **c**, **g** a larger-numerosity vertex per subject (XYZ = coordinates in FreeSurfer fsnative space). These functions are described by a preferred numerosity (mu) and the full width at half maximum (fwhm). **b**, **d**, **f**, **h** Combining neuronal tuning models with a hemodynamic forward model generated predicted time courses (solid lines) for **b**, **d** visual numerosity and **f**, **h** auditory numerosity presentation. Comparing predicted to measured time courses (dotted lines, averaged across runs and cycles) yielded a scaling factor beta quantifying the change in the dependent variable (the hemodynamic signal) and the cross-validated coefficient of determination (cvR²) quantifying the out-of-sample variance explained by the model (calculated across averaged runs).

Building on previous magnetic resonance imaging and electrophysiological work in humans and nonhuman primates, we developed numerosity tuning models describing logarithmic Gaussian functions^4,6^. These models were estimated in individual human subjects, separately for each vertex on the cortical surface. Our models captured the peak response of a neural population to a preferred numerosity and the numerosity range to which a neuronal population responds (full width at half maximum, FWHM) (Fig. 2a, c, e, g; for replication in other subjects, see Supplementary Fig. S1 for visual tuning and Supplementary Fig. S2 for auditory tuning). Summarizing the hemodynamic activation based on these two parameters, these models explained substantial amounts of the observed signal variance related to regional differences in numerosity tuning (visual: maximum cvR² = 0.72; auditory: maximum cvR² = 0.51; both *p* < 0.001) (Fig. 2b, d, f, h; for replication in other subjects, see Supplementary Fig. S1 for visual responses and Supplementary Fig. S2 for auditory responses). Figure 2a–d depicts visual numerosity tuning in vertices from left parietal and occipital cortices. Figure 2e–h depicts auditory numerosity tuning in vertices from right temporal and frontal cortices (see Supplementary Table S2). Hemodynamic signal change was considerably larger in visual maps (up to 3%) than in auditory maps (up to 0.75%) (Fig. 2b, f).

Although the stimulation procedure only included whole numbers (e.g., not three and a half dots), the numerosity tuning model estimates non-integer numerosities for most vertices (Fig. 2). These results were expected since the large neuronal population sampled by each vertex combines nerve cells exhibiting different preferred numerosities. Accordingly, the preferred numerosity of each vertex roughly corresponds to the expected value across all cells.

### Visual and auditory presentation activates distinct numerosity maps

**Fig. 3:**
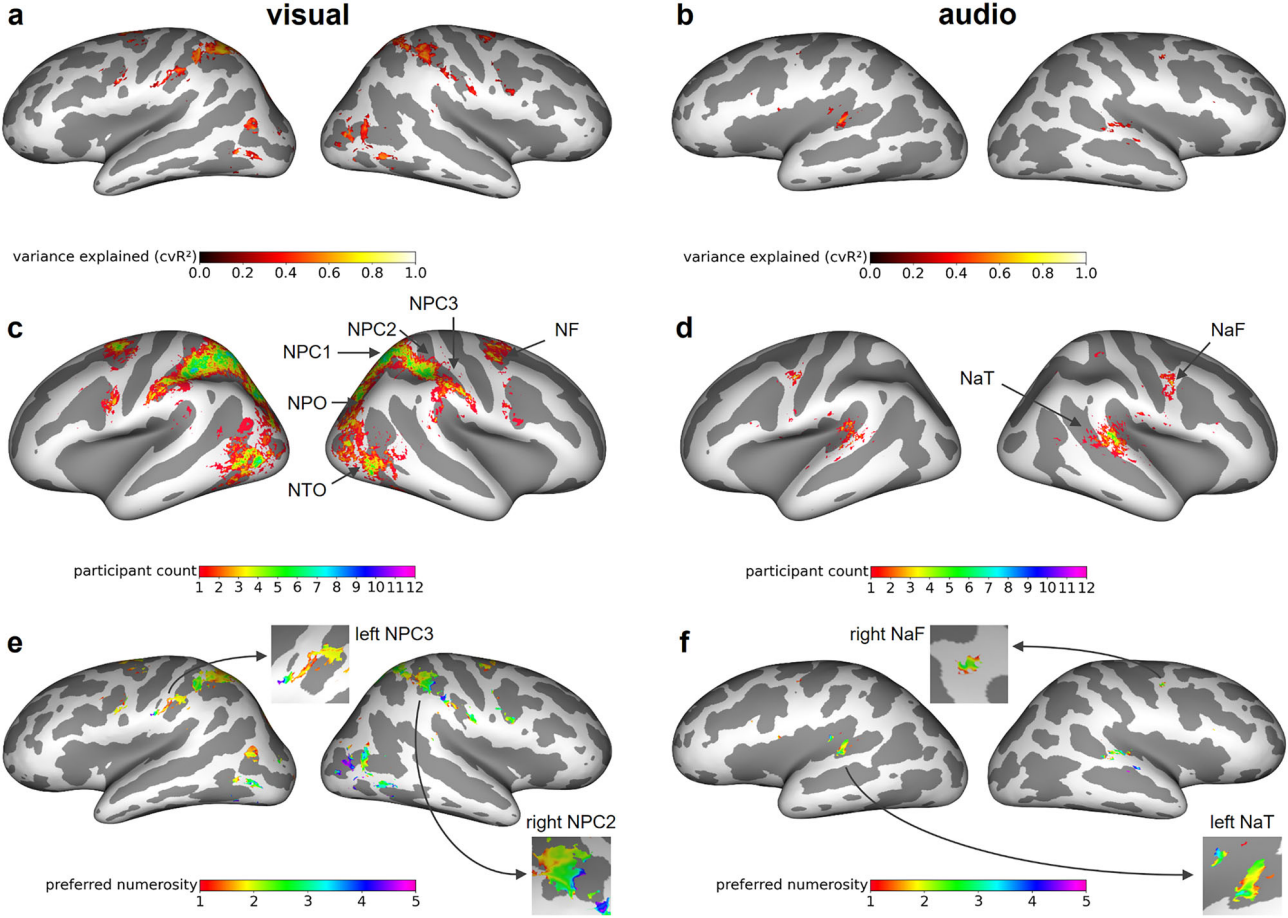
Spatially separated neural responses to visual and auditory numerosity. Inflated surface maps showing regions in which neural tuning models explain a significant amount of the variance for **a** an exemplary subject seeing visual stimuli and **b** another subject hearing auditory stimuli (same subjects as in Fig. 2). The colorbar indicates the variance explained by the model. Participant count maps for **c** visual numerosity and **d** auditory numerosity in standard space (FreeSurfer fsaverage). The colorbar indicates how many subjects exhibit tuning to numerosity according to the variance explained criterion (*p* < 0.05, Bonferroni-corrected). Preferred numerosity maps of single subjects obtained from **e** visual and **f** auditory experiments (same subjects as in **a**, **b**). The colorbar indicates the estimated preferred numerosity. Inset maps show exemplary numerotopic organization in one numerosity map per hemisphere and subject. NTO temporo-occipital visual numerosity field, NPO parieto-occipital visual numerosity field, NPC1,2,3 = parietal visual numerosity fields, NF frontal visual numerosity field, NaT superior temporal auditory numerosity field, NaF superior frontal auditory numerosity field.

**Fig. 4:**
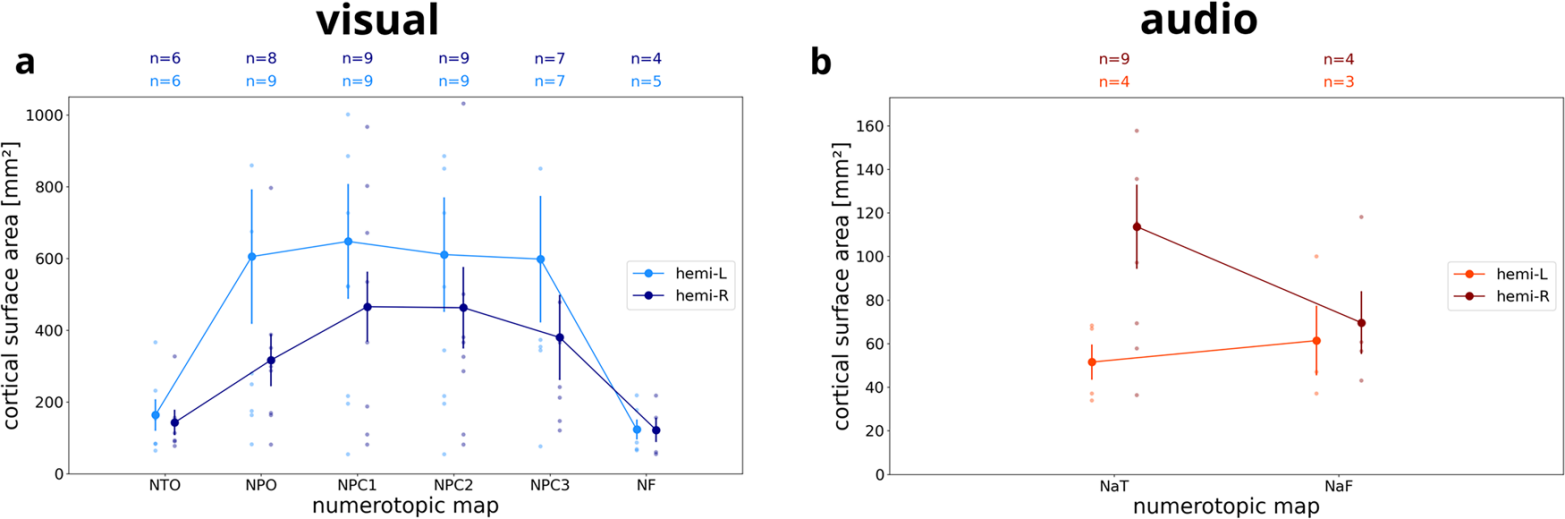
Surface area of numerotopic maps. Cortical surface area differentiated by modality, hemisphere and numerotopic map for **a** visual and **b** auditory numerosity. Plots show means across subjects. Error bars correspond to standard error of the mean. Numbers on top of the graph (n) indicate the number of subjects which exhibit each map, based on minimum cluster size and maximum distance from the center of the map across the group (see Methods). NTO temporo-occipital visual numerosity field, NPO parieto-occipital visual numerosity field, NPC1,2,3 partietal visual numerosity fields, NF frontal visual numerosity field, NaT superior temporal auditory numerosity field, NaF superior frontal auditory numerosity field. L left hemisphere, R right hemisphere.

In each hemisphere, we identified six visual regions and two auditory regions in which the numerosity tuning models explained measured signal variance (Fig. 3a, b; for replication in other subjects, see Supplementary Fig. S3) that was significantly different from zero (*p* < 0.05, Bonferroni-corrected for number of vertices; see Methods). In accordance with the literature^3,5^, we denote these numerosity-selective maps using abbreviations starting with “N” for visual numerosity and starting with “Na” for auditory numerosity. Visual numerosity tuning was evident in temporo-occipital (NTO), parieto-occipital (NPO), posterior parietal (NPC1), dorsal anterior parietal (NPC2), ventral anterior parietal (NPC3) and superior frontal (NF) cortices (Fig. 3c). Auditory numerosity tuning was evident in superior temporal (NaT) and premotor (NaF) cortices (Fig. 3d). None of the regions with numerosity tuning in the visual modality revealed numerosity tuning in the auditory modality and vice versa. All visual numerosity-selective regions, but only the auditory numerosity region NaT were found in a majority of subjects (Fig. 4a, b). In both sensory modalities, these regions revealed considerable individual differences (Fig. 3c, d). Within numerosity-selective regions, vertex-wise preferred numerosities, i.e., stimulus intensities for which vertices revealed peak response, were topographically organized, forming numerotopic maps (Fig. 3e, f; for replication in other subjects, see Supplementary Fig. S4).

### Cortical geometry of visual and auditory maps reveals numerotopic organization

For each single subject, supra-threshold clusters were assigned to numerotopic maps if their cortical surface area was larger than a minimum area (visual: ${A}_{\min }=50\,{{mm}}^{2}$; auditory: ${A}_{\min }=25\,{{mm}}^{2}$) and if their distance from previously reported center coordinates^3^ was smaller than a maximum distance (visual: ${d}_{\max }=25\,{mm}$; auditory: ${d}_{\max }=50\,{mm}$). Analyzing the cortical surface extent of those maps, we found that most numerotopic maps can be identified in the majority of subjects. It turned out that visual numerotopic maps are larger than auditory numerotopic maps by an order of magnitude (Fig. 4a, b).

**Fig. 5:**
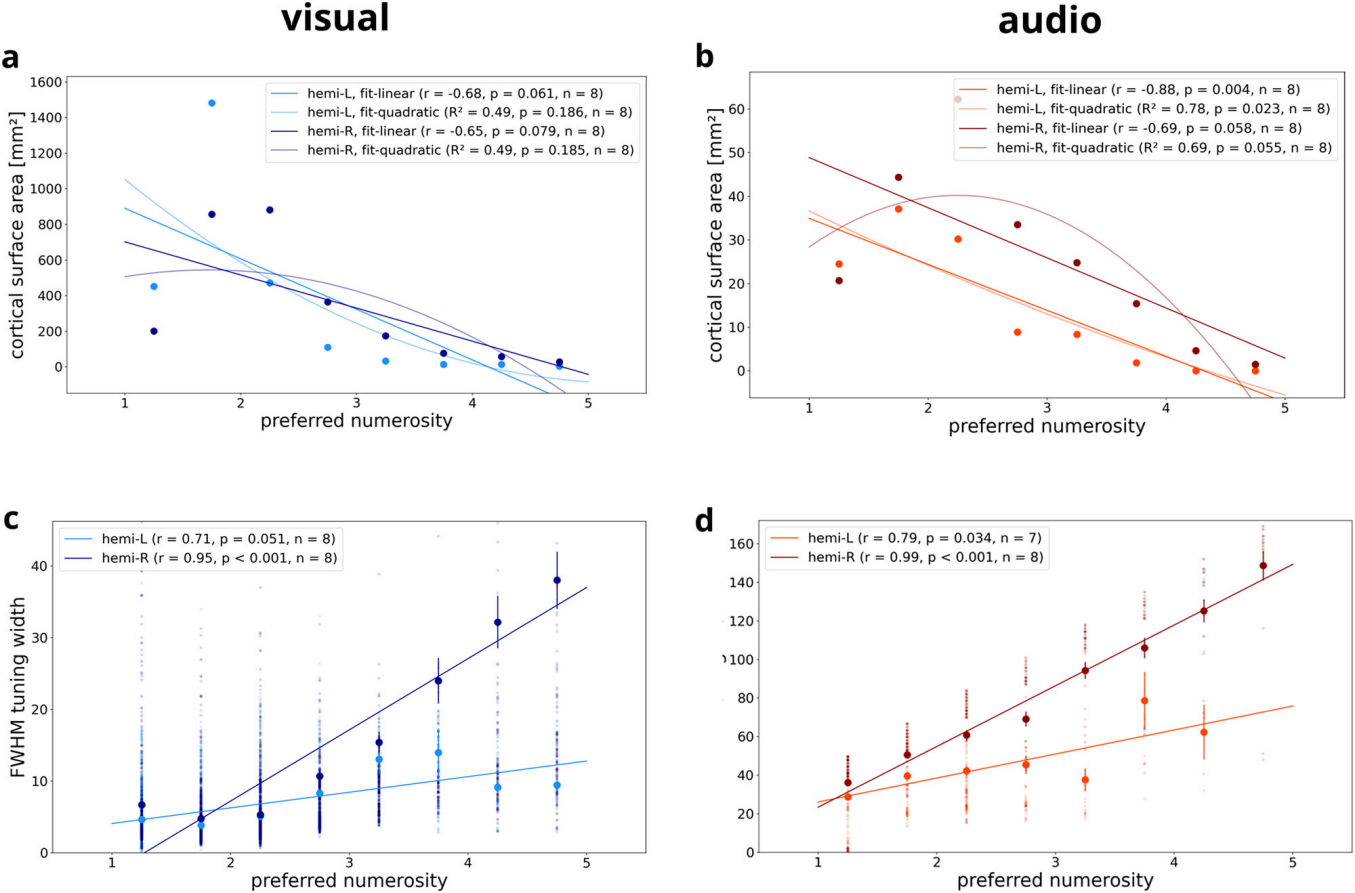
Associations between preferred numerosity, surface area and tuning width. Associations between preferred numerosity and cortical surface area in all supra-threshold vertices for **a** an exemplary subject seeing visual stimuli and **b** another subject hearing auditory stimuli (same subjects as in Figs. 2 and 3). Points correspond to the summed cortical area of all surface triangles of vertices with an average preferred numerosity falling in the respective range (bin width = 0.5). Relationship between preferred numerosity and FWHM tuning width in all supra-threshold vertices for **c** visual numerosity and **d** auditory numerosity (same subjects as in **a**, **b**). Points correspond to mean FWHM in each preferred numerosity bin. Error bars correspond to the standard error of the mean. Solid lines correspond to simple linear regression fits (and fitted quadratic curves in **a**, **b**). L left hemisphere, R right hemisphere. r Pearson correlation coefficient. n number of numerosity bins.

For each hemisphere, we then partitioned the preferred numerosity of all vertices into bins of width 0.5. This resulted in eight bins (1–1.5, 1.5–2, 2–2.5, 2.5–3, 3–3.5, 3.5–4, 4–4.5, 4.5–5) and corresponding bin centers (1.25, 1.75, 2.25, 2.75, 3.25, 3.75, 4.25, 4.75). Then, we calculated how much cortical surface is devoted to representing each level of preferred numerosity. We found a negative correlation between cortical surface area and preferred numerosity for both modalities (Fig. 5a, b; for replication in other subjects, see Supplementary Fig. S5), indicating that fewer cortical surface area represents higher numerosities. Simulations confirmed that, for numerosity-selective voxels, preferred numerosity is accurately estimated by the tuning model, as long as it falls into the presented stimulus range. These results exclude a model bias towards smaller preferred numerosities which could be assumed given that low-numerosity vertices (with a preferred numerosity smaller than around 2.5) were more frequently observed than medium-numerosity vertices (between 2.5 and 5).

We also extracted vertex-wise tuning width, measured as full width at half maximum (FWHM), and analyzed its relationship with binned preferred numerosity in each hemisphere. This analysis revealed a positive correlation between preferred numerosity and FWHM tuning width for both modalities (Fig. 5c, d; for replication in other subjects, see Supplementary Fig. S6), indicating that the larger the numerosity to which a population responds most strongly, the higher the extent to which it also responds to neighboring numerosities.

Next, we ran linear mixed-effects models, using summed cortical surface area or average FWHM tuning width as the dependent variable, binned preferred numerosity and brain hemisphere as independent variables while modeling subject as a random effect. We detected significant negative effects of preferred numerosity on surface area (all *p* < 0.001) and significant positive effects of preferred numerosity on tuning width (all *p* < 0.001) in both hemispheres and modalities (Table 1), indicating that the larger the numerosity, the smaller the cortical surface area covered and the wider the tuning. There were no significant effects of hemisphere on tuning width (visual: *p* = 0.497; auditory: *p* = 0.589), but significant effects on surface area (visual: *p* = 0.065; auditory: *p* < 0.001), with more cortical surface devoted to auditory numerosity in the right hemisphere and more cortical surface devoted to visual numerosity in the left hemisphere (Table 1; also see Fig. 4).

**Table 1:** Associations of surface area and tuning width with preferred numerosity

dependent variable	independent variable	regression coefficient	z-value	p-value	95% CI (lower)	95% CI (upper)
*Session: visual*						
Area	hemi	−156.374	−1.848	0.065	−322.229	9.482
	mu [L]	−118.148	−6.341	<0.001	−154.666	−81.63
	mu [R]	−69.48	−3.728	<0.001	−106.008	−32.953
FWHM	hemi	−3.458	−0.68	0.497	−13.426	6.51
	mu [L]	7.267	6.489	<0.001	5.072	9.461
	mu [R]	8.551	7.634	<0.001	6.355	10.746
*Session: auditory*						
Area	hemi	26.43	4.805	<0.001	15.65	37.211
	mu [L]	−5.798	−4.321	<0.001	−8.428	−3.168
	mu [R]	−11.218	−8.791	<0.001	−13.719	−8.717
FWHM	hemi	−3.87	−0.54	0.589	−17.916	10.176
	mu [L]	11.44	6.58	<0.001	8.032	14.848
	mu [R]	13.388	8.083	<0.001	10.142	16.634

Area cortical surface area measured in mm². *FWHM* tuning width measured as full width at half maximum, *hemi* hemisphere, *mu* preferred numerosity, *CI* confidence interval, *L* left hemisphere, *R* right hemisphere.

**Fig. 6:**
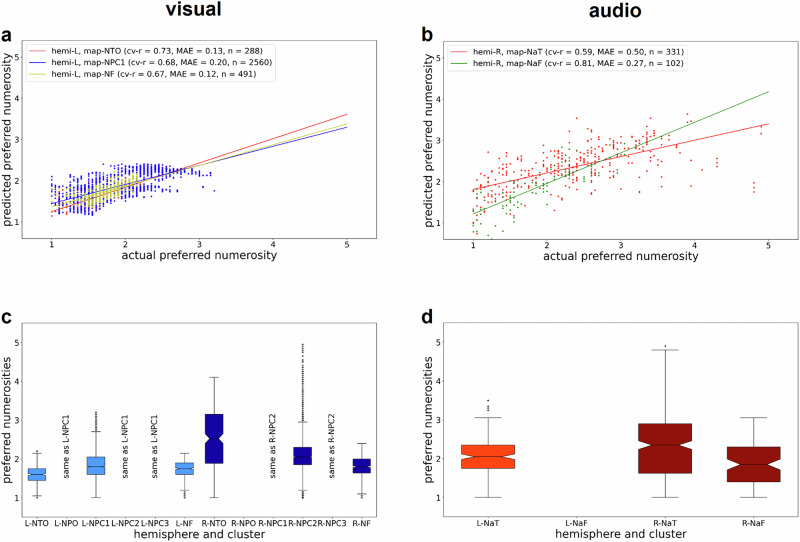
Associations between preferred numerosity and cortical geometry. Preferred numerosity, predicted from cortical surface coordinates, against actual **a** visual and **b** auditory preferred numerosity for numerotopic maps in the left hemisphere for two example subjects (same subjects as in Figs. 2, 3 and 5). Plots show predicted preferred numerosity as a function of estimated preferred numerosity for all vertices belonging to a particular map in standard space. Positive correlations suggest that preferred numerosity can be predicted from surface coordinates, indicating a spatially systematic organization of numerosity selectivity. Ranges of represented numerosity for all observed **c** visual and **d** auditory numerotopic maps of two example subjects. Whenever a cluster covered multiple maps in one hemisphere (e.g., NPC1,2,3), it was assigned to the map the center of which it was closest to. This is denoted in the figure with “same as” clauses for the respective other maps. Boxplots show median and inter-quartile range (IQR). Whiskers extend to the most extreme points lying within 1.5 x IQR from the box. Crosses denote outliers not within 1.5 x IQR from the box.

Previously, topographic organization within numerotopic maps was determined by manually delineating the borders of those maps and correlating average preferred numerosity with normalized cortical distance along the principal axis of each map^3,4^. Here, we instead fitted observed preferred numerosities based on each vertex’s pial surface coordinates in standard space. These models used polynomial expansion of x-, y- and z-coordinates up to order 5 and were estimated with 10-fold cross-validation (see “Methods”). This approach resulted in predicted preferred numerosities which were compared to actual preferred numerosities. For all maps, there were positive correlations between predicted and actual numerosities (Fig. 6a, b; for replication in other subjects, see Supplementary Fig. S7), suggesting relationships between cortical surface coordinates and represented numerosity which, however, are not necessarily linear. For additional analyses in volumetric space accounting for the BOLD point spread function, see Supplementary Fig. S13. Finally, we analyzed the range of numerosities within each numerotopic map on the cortical surface in both hemispheres and for both modalities. The results obtained suggest that not all maps cover the whole range of numerosities presented during the experiment (Fig. 6c, d; for replication in other subjects, see Supplementary Fig. S8).

## Discussion

We identified a common neural population coding scheme underlying visual and auditory quantity detection in the human brain. Populations tuned to numerosity were revealed by electrophysiologically inspired models reconstructing hemodynamic response profiles from high-field magnetic resonance imaging data. These populations were specifically activated by either visual or auditory numerosity and formed anatomically separated numerotopic maps with similar tuning geometries.

The numerosity tuning models used here are based on population receptive field models originally developed for visual cortical responses^7^. Our results go beyond the visual system and demonstrate receptive field-like tuning to auditory numerosities in superior temporal and premotor cortices. Quantity detection thus follows a coherent overarching coding scheme that generalizes across sensory modalities and cortical areas. This coding scheme encompasses temporal and spatial sources of numerical information as well as sequential and simultaneous modes of stimulus presentation. Accordingly, our observation corroborates the vital role of the ability to extract quantitative information from multiple sources of sensory input^5^.

While it is well-established that there are topographically organized responses to visual and tactile numerosity in several cortical regions^3–5^, we show that topographically organized responses to numerosity also exist in human auditory and premotor cortices. Compared to visual maps, auditory maps revealed smaller signal change (Fig. 2f, h), were fewer in number (Fig. 3d), by an order of magnitude smaller (Fig. 4), and larger in the right than in the left hemisphere (Fig. 4b). Moreover, responses to auditory numerosity displayed larger tuning widths compared to visual responses (Fig. 5d), suggesting an inverse relationship between map size and tuning width known from the literature on visual population receptive fields^8^. One possible explanation for these differences between visual and auditory maps might be that it is difficult to subitize auditory numerosity stimuli, unlike in vision or somatosensation^5,9,10^. Another explanation might be that auditory signals could potentially be noisier than visual signals due to residual background scanner sound. This difference would also be consistent with lower response accuracy in auditory versus visual catch trials. Finally, another reason could be that simultaneous numerosity information is instantly accessible while sequential numerosity builds up gradually and stimulus events have to be retained in short-term memory. It also has to be acknowledged that the observed map differences might not be driven by numerosity per se but by extraneous factors, including differences in sensory processing demands, working memory load, attention, or other low-level stimulus properties, such as temporal regularity.

Electrophysiological recordings in frontal and parietal cortices of macaques uncovered single neurons exhibiting tuned responses to the number of sounds in a sequence which the monkeys matched to the numerosity of a subsequently shown visual dot array^6^. Lack of evidence for auditory numerosity tuning in corresponding human cortices might point to a lack of comparability between tasks. Our passive unisensory perception task differs fundamentally from the reward-based working memory-intensive multisensory integration task used in the nonhuman primate study. Other factors that are beyond experimental control relate to further differences between species and recording techniques^3,11^.

The current results suggest that numerosity maps in the left hemisphere occupy larger surface areas but have smaller tuning widths compared to numerosity maps in the right hemisphere. These hemispheric differences are consistent with previous work on visual numerosity maps and extend this principle of lateralization to auditory numerosity maps^3,4^.

In both sensory modalities, numerosity maps revealed pronounced individual differences with respect to sizes, locations, and tuning features. The question of whether these individual neural differences are related to individual cognitive differences in numerical ability cannot be answered based on the current dataset. Follow-up studies tackling this question would have to collect several behavioral measures, like the speed, accuracy, and range of subitizing, and ideally also perform mathematical ability tests in a large sample given the large number of maps and features to be analyzed.

Numerosity co-varies with non-numerical physical features of a stimulus. Varying the number of items in a set changes spatial or temporal features while fixing one feature causes another to vary with the number of items^4^. Accordingly, responses to numerosity should be comparable across systematically varied spatial or temporal features to be considered as abstract^6^. In line with this criterion for abstract representation, responses to visual numerosity have been shown to be equal across co-varying spatial features, such as total area, size, density, circumference and shape^4^. To which degree auditory numerosity responses are robust to co-varying temporal features, such as slower and faster rates and longer and shorter durations, remains to be investigated in future experiments. According to a previous auditory event timing study, superior temporal and premotor cortices respond to rates as fast as about 200 ms (1/frequency) and durations as short as about 100 ms^12^. It can be expected that there are yet to be determined lower and upper thresholds with respect to the rate and duration of presentation, at which stimuli will no longer be perceived as a coherent set of discrete items.

In experiments manipulating temporal features like set durations and intervals, compressive spatial and temporal models combining spatial and temporal population receptive fields outperformed spatial population receptive field models^13–15^. Here we did not vary the temporal profiles to guide the attention of the participants to numerosity detection instead of rhythm perception. The performance of compressive spatial and temporal models could be evaluated in a follow-up experiment including various temporal profiles and a randomized order of numerosities.

It remains an open question how the brain integrates unimodal visual and auditory numerosity information. Integration could be achieved in principle by exchanging information about modality-specific representations or by transforming numerosity information from different sensory sources into a supramodal neural representation, for example in parietal and frontal cortices^16,17^. The present fMRI findings do not support the notion of a supramodal numerosity processing network but rather the notion of an interactive modality-specific numerosity processing network. However, future studies employing multi-unit electrophysiology and stimulation techniques are needed to test these mutually non-exclusive hypotheses.

To date, fMRI studies on numerosity perception have exclusively focused on young healthy adult participants. How numerotopic maps emerge in the course of brain development is currently unknown. It is possible that these maps already form in the first months of life given the behavioral evidence for numerosity discrimination in infants^18^. At the same time, the observation that numerosity discrimination ability refines substantially towards adulthood suggests that numerotopic maps may undergo developmental reorganization, such as increasingly sharper tuning^19^. These mutually non-exclusive predictions remain to be tested in future longitudinal studies with pediatric samples.

## Methods

Parts of the text in the methods section are cited from a methodologically related article previously published by the authors^20^.

### Participants

Following previous electrophysiological and magnetic resonance imaging work, we employed a single-subject design focused on individual data sets^1–4^. Twelve human participants (aged 20–34 years, all right-handed, six males; see Supplementary Table S1) were recruited to demonstrate reproducibility and generalization across subjects. Given that the datasets were in agreement and led to similar conclusions with high statistical confidence, we did not consider it as scientifically necessary, economically and ecologically reasonable and ethically plausible to include more participants. All participants had normal or corrected to normal visual acuity, normal hearing ability and were well educated (eleven college degrees, one vocational degree). All participants with corrected to normal visual acuity wore contact lenses or had undergone laser eye treatment. Normal hearing acuity was determined based on available medical records (e.g., audiometry performed for getting a driver’s license). All participants gave written informed consent to participate. All ethical regulations relevant to human research participants were followed. The study was approved under the reference 317/19-ek by the Ethics Committee at the Medical Faculty of the University of Leipzig, Germany (IRB00001750).

### Sensory stimulation

#### Visual stimuli

In the visual experiment, stimuli were projected on a 34 × 25 cm screen inside the MRI bore with a resolution of 1024 × 768 pixels. Participants viewed this screen through a mirror attached to the head coil. Stimuli were black dots on a middle gray background presented at the center of the display. To guide fixation and maintain attention, dots appeared within a radius of 0.75° (visual angle) of a diagonal cross of thin red lines covering the entire display throughout the experiment. To control for low-level non-numerical visual features, summed surface area (and thus luminance) was kept constant and dots were distributed homogeneously (preventing perceptual grouping) while randomly varying the positions of the dots. All stimuli were created using PsychoPy version 2021.2.3^21^. Other low-level features have been repeatedly and consistently shown not to influence numerosity-specific hemodynamic time courses^3,4^.

#### Auditory stimuli

In the auditory experiment, participants listened to beep tone sequences via magnetic resonance compatible MR Confon headphones (http://www.mr-confon.de/) at an individually comfortable sound pressure level. To control for low-level non-numerical auditory features, the auditory frequency (pitch) within each sequence was randomly varied between 333, 359, 392 and 440 kHz. All stimuli were created using Audacity 3.0.2 (https://www.audacity.de/).

#### Stimulation procedure

Numerosities one through five were used to capture both the subitizing range (one through three) and the estimation range (four to five)^22^. Our rationale for restricting the stimulus set to the range of 1 to 5 was that previous work suggested that topographic visual numerosity maps with logarithmic Gaussian tuning cover both small numerosities (1–7) and large numerosities (8, 16, 32, 64)^22^. Furthermore, we aspired to keep the total duration of each experiment as short as possible given that framewise displacement increases with acquisition time and compromises data quality^23^.

Numerosities were first presented in ascending order in blocks changing every 4200 ms. Within each block of the visual experiment, a dot array was shown six times for 300 ms each, alternating with 400 ms of gray background. Within each block of the auditory experiment, a tone sequence was played six times for 500 ms each, alternating with 200 ms of silence. The duration of a single tone was kept constant at 60 ms and the duration of the following silence period was kept constant at 40 ms within a tone sequence. For example, in a tone sequence corresponding to the numerosity three, 60 ms of tone and 40 ms of silence were presented thrice at the beginning of a stimulation period of 500 ms. Short stimulus durations were chosen to prevent participants from counting and to avoid adaptation effects. Short equal pauses were introduced to prevent participants from keeping numerosities in working memory. After the ascending numerosity sequence, twenty items (visual dots and audio beeps, respectively) were presented 24 times in the visual experiment and 8 times in the auditory experiment for 16.8 s including pauses. Next, numerosities one through five were presented again, but in descending order, followed by another block of twenty items. This stimulation cycle (1 – 2 – 3 – 4 – 5 – 20 – 5 – 4 – 3 – 2 – 1 – 20) was repeated four times in each run. The rationale for the long baseline period was that hemodynamic responses could return to baseline for numerosity-selective regions with small preferred numerosities and little neural responses to 20 items. The rationale for the ascending-descending numerosity presentation was to avoid adaptation effects, because each stimulus is preceded by both lower and higher numerosities in different blocks of presentation, thereby counterbalancing potential effects of previously presented numerosity^4^. Additionally, keeping this fixed and smooth order of presentation is known to result in more homogeneous topographic maps than a pseudorandom stimulation sequence^24^. Alternating between ascending and descending presentation served to minimize neural adaptation by ensuring each stimulus was preceded by a stimulus inducing both a lower and higher response^4^. Stimuli were presented with PsychoPy version 2021.2.3^21^. There were eight runs in each session (visual and auditory) for a total experiment duration of 41 min per session.

#### Target detection task

In 10% of all stimulus events, white instead of black dots were shown during the visual experiment and 1000 kHz beeps were played instead of 333 to 440 kHz beeps during the auditory experiment. Participants were instructed to respond to these events by pressing a button. This simple target detection task was introduced to ensure participants were paying attention and following the task.

### Data acquisition

#### Magnetic resonance imaging

T2*-weighted functional magnetic resonance imaging data were acquired with a 1-channel transmit/32-channel receive coil (NOVA Medical, Wilmington MA, USA) on a 7 Tesla Magnetom TERRA scanner (Siemens Healthineers, Erlangen, Germany). We used a 2D gradient-echo echo-planar imaging (2D-EPI) sequence for acquiring 41 slices (thickness = 1.75 mm) with a field of view FOV = 192 mm × 192 mm and a matrix size = 110 × 110 resulting in an isotropic voxel size of 1.75 mm × 1.75 mm × 1.75 mm. The slice package had a transversal orientation, but was tilted towards coronal orientation in order to spare anterior frontal and temporal lobes where pronounced B0 and B1 inhomogeneities degrade image quality at 7 T. To achieve a repetition time TR of 2.1 s, the acquisition was accelerated using GRAPPA with an iPAT factor of 3. An echo time TE of 24 ms, a flip angle FA of 70° and a receiver bandwidth of 1684 Hz/Px was used. 145 volumes were acquired per run resulting in a single run time of 05:04.50 min. For distortion correction, an EPI data set using the same parameters, but with the phase-encoding direction flipped from anterior-posterior to posterior-anterior was acquired. For anatomical referencing, a T1-weighted image was acquired using an MP2RAGE sequence^25^ with the following parameters: TR = 5.000 ms, TE = 2.01 ms, TI_1/2_ = 900 ms/2.750 ms, FA_1/2_ = 5°/3°, isotropic voxel size of 0.7 mm × 0.7 mm × 0.7 mm.

### Data preprocessing

Preprocessing was performed using FreeSurfer 7.4.1^26^ and fMRIprep 23.1.4^27^, based on Nipype 1.8.6^28^. The following description of fMRIPrep’s preprocessing is based on a boilerplate distributed with the software covered by a ‘no rights reserved’ (CC0) license. For more details about the pipeline, see the section corresponding to each workflow in the fMRIPrep documentation.

#### Structural preprocessing

Before running fMRIPrep, we segmented the MP2RAGE UNI images using a custom workflow implemented in the python package fmritools 1.0.3 (10.5281/zenodo.10573042). MP2RAGE image backgrounds were denoised as described in O’Brien et al.^29^ and the resulting images were bias-corrected with SPM12. Then the first five Freesurfer cortical reconstruction steps were applied before computing a skullstrip mask based on the INV2 of the MP2RAGE image. This skullstrip mask was applied to the remaining Freesurfer cortical reconstruction pipeline. As a first step in fMRIPrep, the denoised and thresholded structural images were corrected for intensity non-uniformity (INU) with N4BiasFieldCorrection^30^, distributed with ANTs^31^. Images were then skull-stripped with a Nipype implementation of the antsBrainExtraction.sh workflow (implemented in ANTs), using OASIS30ANTs as target template. Brain tissue segmentation of cerebrospinal fluid (CSF), white-matter (WM) and gray-matter (GM) was performed on the brain-extracted images using FSL FAST^32^. In the case that two T1w images were available for a subject, a structural T1w reference map was computed after co-registration of the INU-corrected T1w images using mri_robust_template implemented in FreeSurfer 7.3.2^33^. Otherwise, the INU-corrected T1w image was used as T1w reference throughout the pipeline. Brain surfaces were reconstructed using the recon-all procedure of FreeSurfer 7.3.2^26^. Brain masks estimated before were refined based on Mindboggle’s method to integrate ANTs-derived and FreeSurfer-derived segmentations of the cortical gray matter. This way, the best results of the ANTs-derived and FreeSurfer-derived cortical gray-matter segmentations could be included in the final masks^34^.

#### Functional preprocessing

Before functional data preprocessing, image quality was assessed using MRIQC and high-motion runs (framewise displacement >1.7 mm for >10% of volumes) were excluded from further analysis^35^. Using fMRIPrep 23.1.4, for each of the up to 16 functional runs per subject (across both tasks), the following preprocessing steps were taken: First, a reference volume for estimating head-motion parameters and its skull-stripped version were generated. Head motion parameters (transformation matrices, and six corresponding rotation and translation parameters) were estimated before any spatiotemporal filtering using FSL MCFlirt^36^. B0-nonuniformity maps (fieldmaps) were generated from two (or more) EPI references with FSL topup^37^. Fieldmaps were then aligned with rigid registration to the target EPI reference run. Field coefficients were mapped onto the reference EPI using the transformation matrices generated when aligning the fieldmaps with the reference EPI. Next, functional runs were slice-time-corrected to 1.025 s (which is equivalent to 0.5 of the slice acquisition range of 0-2.05 s) using AFNI’s 3dTshift^38^.

Functional reference images were then co-registered to the T1w reference images by applying boundary-based registration with bbregister in FreeSurfer^39^. Co-registration was configured with six degrees of freedom. Several confounding time-series were calculated based on the preprocessed functional images: framewise displacement (FD), DVARS (D referring to temporal derivative of time courses, VARS referring to root mean square variance over voxels) and three region-wise global mean signals extracted from within the CSF, the WM, and the whole-brain masks. FD was computed using two formulations based on the absolute sum of relative motions and the relative root mean square displacement between affines^37,40^. FD and DVARS were calculated for each functional run using their implementations in Nipype which follow the definitions by Power and colleagues^40^.

These nuisance variables and head motion estimates were returned as a confounds file for each individual run. Specifically, the confound time series derived from head motion estimates and global signals were expanded by including temporal derivatives and quadratic terms for each^41^. Frames that exceeded a threshold of 1.7 mm FD or 1.5 standardized DVARS were annotated as motion outliers. Additional nuisance time series were calculated by performing principal components analysis for the signals found within a thin band (crown) of voxels around the edge of the brain, as proposed by Patriat and colleagues^42^.

The functional time-series were resampled onto the fsnative (individual participant) and fsaverage (standard space) surfaces following the FreeSurfer reconstruction nomenclature. All resamplings were performed with a single interpolation step by composing all the pertinent transformations (i.e., head-motion transform matrices, susceptibility distortion correction if available, and co-registrations to anatomical and FreeSurfer output spaces). Non-gridded (surface) resamplings were performed using mri_vol2surf in FreeSurfer. No spatial or temporal smoothing was applied^43^.

### Statistics and reproducibility

A population receptive field model describing neural and hemodynamic responses to presented numerosity was fitted to each individual vertex in a mass-univariate fashion. Estimated parameters from this model (preferred numerosity, tuning widths, scaling factor) were extracted from this model and submitted to analyses across vertices and subjects. At the single-subject level, statistical significance of numerosity selectivity was determined by comparing the receptive field model against a null model not accounting for presented numerosity. Extracted parameters were submitted to correlational analyses to identify relationships with cortical geometry. Linear mixed-effects models with subject as random effect were estimated to test effects of preferred numerosity on tuning width and cortical surface area at the group level.

No sample-size calculation was performed. Our analysis is based on 24 datasets collected from 12 participants. The main experimental effects of interest were analyzed within each participant (across 8 runs, each including 4 cycles, each including 6 different numerosity levels in repeated presentations) and replicated across 12 independent participants.

### Numerosity analysis

#### Numerosity population receptive field models

Task-based fMRI data were analyzed using a well-established forward modeling approach^3–5,7,44^. Each vertex is thought to represent the presented numerosities using a logarithmic Gaussian tuning curve characterized by two parameters: the preferred numerosity $\mu$ (the numerosity to which the population responds strongest) and a tuning width $\omega$ (the full width at half maximum (FWHM) of the response function). Given presented numerosity ${x}_{t}$ at continuous time $t$, the neuronal response at $t$ is given by

$${z}_{t}=exp \left[-\frac{1}{2}{\left(\frac{{ln}\,{x}_{t}-{mu }_{log }}{{\sigma }_{log }}\right)}^{2}\right]$$

where ${\mu }_{\log }={\mathrm{ln}}\mu$ and ${\sigma }_{\log }$ is the standard deviation of the response function in logarithmic numerosity space from which the FWHM tuning width in linear numerosity space can be calculated:

$$\omega =\exp \left[{\mu }_{\log }+\sqrt{2{\mathrm{ln}}2}{\sigma }_{\log }\right]-\exp \left[{\mu }_{\log }-\sqrt{2{\mathrm{ln}}2}{\sigma }_{\log }\right]$$

Given an assumed hemodynamic response function (HRF) $h(t)$, the hemodynamic response is given by the convolution of the neuronal response with the HRF:

$${s}_{t}={z}_{t}* h(t)$$

Finally, a linear relationship is assumed between this hypothesized hemodynamic response, governed by the neural tuning parameters $\mu$ and $\omega$, and the preprocessed fMRI signal

$${y}_{t}=\beta \cdot {s}_{t}+{\beta }_{0}+{\varepsilon }_{t}$$

where $\beta$ is a scaling factor of the hypothesized signal, ${\beta }_{0}$ is the baseline level of hemodynamic activity and ${\varepsilon }_{t}$ are noise terms independent and identically distributed (i.i.d.) with zero mean and unknown variance. Before estimation of tuning parameters $\mu$, $\omega$ and $\beta$, preprocessed fMRI signals were standardized to units of percent signal change and 12 confound variables were regressed from standardized signals (six motion parameters; WM, CSF and global signal; three cosine regressors modeling temporal drifts). Then, signals were averaged across runs (but not cycles) and numerosity analysis was performed for a single averaged run.

#### Estimation of numerosity tuning parameters

The numerosity pRF model defines a likelihood function of the measured data $y$, given the tuning parameters $\mu$ and $\omega$. Some combinations of tuning parameters make the measured signals more likely than others and the goal is to find the tuning parameters which are most compatible with the measured fMRI data. To this end, we specified a large grid of plausible values: $\mu$ from 0.8 to 5.2 in steps of 0.05, ${\sigma }_{\log }$ from 0.05 to 30 in steps of 0.05 (such that $0.12\le \omega \le 34.17$ for the lowest presented numerosity $\mu =1$ and $0.59\le \omega \le 170.9$ for the highest presented numerosity $\mu =5$; cf. equation above). For each possible combination $\mu$ and $\omega$, the ensuing neuronal responses $z$ were calculated using the sequence of presented numerosities $x$ and the ensuing hemodynamic signals $s$ were generated using the canonical HRF, as implemented in SPM. Then, $\beta$ was estimated from the measured fMRI signal $y$ using ordinary least squares, resulting in a single log-likelihood value for each combination of $\mu$ and $\omega$:

$${LL}\left(\mu ,\omega \right)={ln}\,p\left(y|\mu ,\omega ,\hat{\beta }\right)$$

Finally, the optimal tuning parameters are those which maximize the log-likelihood function across all possible combinations:

$$\left(\hat{\mu },\hat{\omega }\right)={argmax}\,{LL}\left(\mu ,\omega \right)$$

When not accounting for serial correlation in fMRI signals, this is equivalent to estimating tuning parameters by minimizing the residual sum of squares (RSS), as was done previously^4,7^.

#### Cross-validated estimation of model fit

For unbiased estimation of tuning model precision, we applied split-half cross-validation and partitioned recorded fMRI data into odd runs (runs 1, 3, 5, 7) and even runs (runs 2, 4, 6, 8) for each participant. Then, tuning parameters $\mu$ and $\omega$ were estimated from one half of the data (averaged over runs) and used to generate expected hemodynamic time courses. These time courses were then compared against the measured signals from the other half of the data (averaged over runs), yielding a coefficient of determination (${R}^{2}$). Cross-validated model fit was obtained by averaging coefficients of determination from odd and even runs. This is referred to as cvR² and was used as the primary criterion for selecting supra-threshold vertices.

#### Considered, but suboptimal, hence unused analysis options

The above procedure for parameter estimation via maximum likelihood is equivalent to ordinary least squares under i.i.d. errors and no serial correlations. We did not account for serial correlations using e.g., restricted maximum likelihood and weighted least squares, since this did not improve tuning parameter estimates in a pre-analysis simulation study, even if serial correlations were present in the simulated signals. We also did not include hemodynamic derivatives to account for possible variation of HRF between subjects and vertices, because this was found to generate rather noisy numerosity-selective clusters on the cortical surface in a single-subject pilot analysis.

Finally, we also tested linear tuning functions in which the neuronal response at $t$ is given by

$${z}_{t}=exp \left[-\frac{1}{2}{\left(\frac{{x}_{t}-{\mu }_{{lin}}}{{\sigma }_{{lin}}}\right)}^{2}\right]$$

where ${\mu }_{{lin}}=\mu$ and ${\sigma }_{{lin}}$ is the standard deviation of the response function in linear numerosity space from which the FWHM tuning width can be calculated:

$$\omega =2\sqrt{2\,{ln}\,2}{\sigma }_{{lin}}$$

Comparing cross-validated variance explained (cvR²) between linear and logarithmic tuning models, pooled across subjects, we found systematic advantages of logarithmic tuning functions in numerosity-selective vertices in both hemispheres and both modalities (see Supplementary Fig. S9). In addition, linear tuning models lead to dubious results, with maximum-cvR² vertices merely reproducing differences between numerosities 1–5 and 20, rather than showing actual numerosity tuning in the small-numerosity range and instead displaying nearly flat tuning functions (see Supplementary Fig. S10). Consequently, we only report analyses with logarithmic tuning functions.

#### Control analysis against categorical numerosity effect

In order to rule out the possibility that neuronal populations merely react to the categorical difference between small numerosities (1–5) and the large numerosity (20), we implemented a vertex-wise classical GLM analysis. At the single-subject level, a categorical model with six HRF regressors for numerosities 1, 2, 3, 4, 5, 20 and twelve confound variables (same as for numerosity pRF analysis; see above) was specified and estimated. For each subject, a contrast map “1–5 minus 20” was calculated using the contrast vector $c=\left[\right.\frac{1}{5},\frac{1}{5},\frac{1}{5},\frac{1}{5},\frac{1}{5},$ −*1*]. At the group level, a one-sample t-test was run across contrast maps from all subjects and statistical inference was performed using t-contrasts for positive effects (1–5 > 20) and negative effects (20 > 1–5) of small numerosities for both modalities, visual and auditory (see Supplementary Fig. S11).

Except for some parietal clusters in the visual experiment, those effects were restricted to higher responses for the large numerosity (20 > 1–5). However, observing such effects when using the numerosity tuning model would require either a preferred numerosity close to this stimulus level ($\mu \approx 20$) or a small preferred numerosity along with negative scaling factor ($\beta < 0$). Since both are filtered out when selecting numerosity-selective vertices (requiring positive scaling factor and numerosity between 1 and 5; see below), our results are unlikely to reflect categorical differences between small and large numerosities. Moreover, theoretical simulation analyses indicate that differential responses to numerosities can emerge even with high tuning width (e.g., fwhm $\approx$ 80) for both, low and medium preferred numerosities (see Supplementary Fig. S12).

### Statistical analyses

#### Extraction of parameter estimates

The numerosity pRF model returns four quantities for each vertex: estimated preferred numerosity$\,\hat{\mu }$, estimated FWHM tuning width $\hat{\omega }$, estimated scaling factor $\hat{\beta }$ and cross-validated ${R}^{2}$ (cvR²). Only vertices that exhibited a positive scaling factor ($\hat{\beta } > 0$), an estimated preferred numerosity inside the presented stimulus range ($1\le \hat{\mu }\le 5$) and statistically significant variance explained (*p* < 0.05, Bonferroni-corrected; see below) were included in the following analyses. The rationale for excluding vertices with negative scaling factor was that the preferred numerosity could not be interpreted as the stimulus generating the maximum response. The rationale for excluding vertices with preferred numerosity outside the stimulus range is that their tuning functions will be monotonically increasing or decreasing throughout the stimulus range and thus not display actual tuning. Simulations indicate that numerosity-selective voxels can be distinguished from no-signal voxels with high sensitivity ($\approx$ 85%) and specificity ($\approx$ 100%) and that preferred numerosity is accurately estimated, if it falls into the presented stimulus range (https://github.com/SkeideLab/EMPRISE-analysis/blob/main/code/Python/Demo.pdf).

#### Inferential statistics for variance explained

To assess statistical significance of variance explanation by the tuning model, we performed an F-test of the model including the numerosity regressor (generated using tuning parameters obtained from the other half of the data) against a null model including only the baseline regressor:

$$F=\frac{\left({{RSS}}_{0}-{RSS}\right)/\left(p-1\right)}{{RSS}/\left(n-p\right)}$$

where $n=145$ is the number of scans per run and $p=2$ is the number of free parameters in the numerosity pRF model (${\beta }_{0},\beta$). Note that $\mu$ and $\omega$ were not counted into the number of free parameters, because they were estimated from independent data (the respective other half of the fMRI runs). Under the null hypothesis ${H}_{0}:{R}^{2}=0$, this test statistic is following an F-distribution with $p-1$ numerator and $n-p$ denominator degrees of freedom. The F-statistic can also be expressed in terms of (cross-validated) variance explained:

$$F=\frac{{R}^{2}/\left(p-1\right)}{\left(1-{R}^{2}\right)/\left(n-p\right)}$$

Based on this, cvR² maps were thresholded by applying a significance level of $\alpha =0.05$, Bonferroni-corrected for the number of vertices in the current hemisphere. For example, with 100,000 vertices per hemisphere (actual numbers ranged between 90,915 and 116,566 in native subject space), this implies a variance explained threshold of ${R}^{2}=0.1625$. Only vertices surpassing this threshold (derived from $n$, $p$, $\alpha$ and number of vertices) were retained for the following analyses.

#### Calculation of cortical surface area

Alongside with estimated tuning parameters, the coordinates on the pial surface in native subject space were extracted for each supra-threshold vertex. We here chose pia mater rather than white matter or midthickness surface coordinates, as these most precisely correspond to actual cortical surface extents in native subject space. After filtering numerosity maps with the criteria mentioned above, AFNI’s SurfClust function was used to form clusters of vertices with an edge distance of at most 1 on the cortical surface (i.e., vertices needed to be directly connected to belong to the same cluster). For each cluster, the total area on the cortical surface was calculated as the summed area of all triangles for which all three nodes belonged to the cluster. For analyses of cortical geometry, only clusters having a minimum cortical surface area (visual: ${A}_{\min }=50\,{{mm}}^{2}$; auditory: ${A}_{\min }=25\,{{mm}}^{2}$) were retained.

#### Identification of numerotopic maps

In order to investigate topographic organization in larger numerosity-selective clusters, supra-threshold clusters were assigned one of the previously reported six numerotopic maps^3^ for the visual modality (temporo-occipital, NTO; parieto-occipital, NPO; in parietal cortex, NPC1/2/3; in prefrontal cortex, NF; N = numerosity) by being in less than some distance (${d}_{\max }=25\,{mm}$) from previously reported center coordinates^3^ with at least one vertex in standard space. For the auditory modality, two numerotopic maps were identified from participant count maps as being located in primary auditory cortex (temporal, NaT) and pre-motor regions (frontal, NaF; Na = auditory numerosity)^5^ and clusters were assigned to these auditory numerosity maps in the same way (${d}_{\max }=50\,{mm}$).

#### Visualization of numerosity selectivity

The estimated tuning parameters $\hat{\mu }$ and $\hat{\omega }$ describe a logarithmic Gaussian tuning function and an expected time course of hemodynamic activity in a single cycle of numerosity presentation. These are plotted for a low-numerosity vertex ($1\le \hat{\mu }\le 2$) and a high-numerosity vertex (visual: $4\le \hat{\mu }\le 5$; auditory: $3\le \hat{\mu }\le 5$) from exemplary single subjects in each sensory modality (see Fig. 2 and Supplementary Figs. S1/S2). In addition to this, vertex-wise variance explained and vertex-wise preferred numerosity are visualized in native subject space for all vertices passing the above filtering criteria (see Fig. 3a, b, e, f and Supplementary Figs. S3/S4). For each vertex in standard space, the number of subjects showing numerosity selectivity according to these criteria are shown as participant count maps (see Fig. 3c, d).

#### Statistical analysis of cortical geometry

For each each numerotopic map (six visual, two auditory; see above) in each hemisphere (left, right), we report the average cortical surface area (see Fig. 4) as well as the represented range of preferred numerosities (see Fig. 6c, d and Supplementary Fig. S8). Additionally, we investigated whether there is a topographic organization within those maps, i.e., progression of preferred numerosity along the cortical surface. Previously, this was done by manually delineating start, end and borders of numerotopic maps, establishing distance along the so-defined principal axis of a numerotopic map and calculating average preferred numerosity as a function of binned cortical distance^3,4^. As manual delineation was not feasible for our amount of data (twelve participants, two sessions, six/two maps) and might lead to non-reproducible results, we instead used a linear regression model with preferred numerosity as the dependent variable and pial x-, y- and z-coordinates in standard space as independent variables:

$${\mu }_{x,y,z}={\beta }_{0}+\mathop{\sum }\limits_{k=1}^{d}({\beta }_{x,k}\cdot {x}^{k}+{\beta }_{y,k}\cdot {y}^{k}+{\beta }_{z,k}\cdot {z}^{k})+{\varepsilon }_{x,y,z}$$

where $x$, $y$ and $z$ refer to mean-centered coordinates of one vertex inside the cluster and $d=5$ is the order of polynomial expansion. This model was estimated using 10-fold cross-validation, predicting preferred numerosity in the left-out set of vertices. Predictive performance is assessed by calculating actual and predicted preferred numerosity (cv-r) as well as mean absolute error (MAE) (see Fig. 6a, b and Supplementary Fig. S7). If there is a significant correlation between actual numerosities and predicted numerosities, this indicates that preferred numerosity changes along spatial dimensions and is thus topographically organized.

#### Statistical analysis of tuning parameters

As further correlational analyses, attempting to replicate previously reported effects^3,4^, we analyzed relationships of preferred numerosity with cortical surface area (see Fig. 5a, b and Supplementary Fig. S5) and FWHM tuning width (see Fig. 5c, d and Supplementary Fig. S6) for each hemisphere. Additionally, we ran a linear mixed-effects model with either cortical surface area or FWHM tuning width as the dependent variable, preferred numerosity and hemisphere as independent variables and subject as a random effect (see Table 1).

### Reporting summary

Further information on research design is available in the Nature Portfolio Reporting Summary linked to this article.

## Supplementary information

Supplementary Information

Reporting Summary

## Data Availability

The data for this study is available through a public link on Edmond – the Open Research Data Repository of the Max Planck Society: 10.17617/3.PXTX9U^45^.

## Code availability

All data analysis was performed with Spyder 5.4.3 using Python 3.10 and various third-party packages (numpy 1.24.3, scipy 1.10.1, pandas 2.1.1, statsmodels 0.14.0, nibabel 5.1.0), except for edge-based surface clustering which was run using AFNI’s SurfClust. Brain surfaces were visualized with surfplot 0.2.0 and other plots were generated with matplotlib 3.7.1. All code for data analysis is available from GitHub (https://github.com/SkeideLab/EMPRISE-analysis).
