# Supplementary material for: Population coding for visual and auditory quantity in human numerotopic maps
Source: Commun Biol. 2026 Feb 27;9:383. doi: 10.1038/s42003-026-09752-2 (PMC12992664; doi:10.1038/s42003-026-09752-2)
Supplement: Supplementary file 1 — Supplementary Information [file 42003_2026_9752_MOESM1_ESM.pdf]

– Supplementary Online Material –

Population coding for visual and auditory quantity in human numerotopic maps

Garam Jeong[1]*, Joram Soch[2]*, Robert Trampel[3], Andreas Nieder[4] and Michael A. Skeide[1,5] ✉

[1] Research Group Learning in Early Childhood,

Max Planck Institute for Human Cognitive and Brain Sciences,

Stephanstraße 1A, 04103 Leipzig, Germany

[2] Institute for Psychology,

Otto-von-Guericke-Universität Magdeburg

Universitätsplatz 2, 39106 Magdeburg, Germany

[3] Department of Neurophysics,

Max Planck Institute for Human Cognitive and Brain Sciences,

Stephanstraße 1A, 04103 Leipzig, Germany

[4] Animal Physiology Unit, Institute of Neurobiology,

Eberhard-Karls-Universität Tübingen,

Auf der Morgenstelle 28, 72076 Tübingen, Germany

[5] Institute of Child and Adolescent Psychiatry,

Christian-Albrechts-Universität zu Kiel,

Niemannsweg 147, 24105 Kiel, Germany

*These authors contributed equally: Garam Jeong, Joram Soch.

✉ e-mail: skeide@cbs.mpg.de

Correspondence should be addressed to Michael A. Skeide

Supplementary Tables

subject ID	gender	age	visual hit rate	auditory hit rate
001	F	27.5	99.42	78.75
002	M	26	99.42	80.42
003	M	22	99.42	79.58
004	F	30.5	100.00	78.75
005	F	24.5	99.13	83.33
006	M	20	99.42	77.08
007	F	20	99.71	80.00
008	M	27	100.00	83.33
009	F	22	99.71	79.58
010	M	22	100.00	81.67
011	F	34	100.00	81.67
012	M	33.5	100.00	80.42

Table S1: Participant details. Pseudonymous subject ID, gender (F = female, M = male) and age in years (broken number when age changed across scanning sessions) as well as catch trial hit rates for the two modalities in percent (averaged across runs).

Figure	tuning parameters	native coordinates	standard coordinates	anatomical location (AAL regions)
2a/b	mu = 1.80, fwhm = 3.31	[-18, -26, 55]	[-29, -52, 46]	Parietal_Inf_L (NPC1)
2c/d	mu = 4.40, fwhm = 19.4	[-46, -55, 7]	[-44, -83, -4]	Occipital_Inf_L (NTO)
2e/f	mu = 1.40, fwhm = 37.8	[46, 11, 61]	[46, 2, 48]	Precentral_R (NaF)
2g/h	mu = 3.35, fwhm = 20.2	[64, -8, 26]	[68, -31, 12]	Temporal_Sup_R (NaT)

Table S2: Anatomical locations. Automated anatomical labeling (AAL) regions for vertices reported in Figure 2. Visual vertices are in the left hemisphere, auditory vertices are in the right hemisphere.

Supplementary Figures

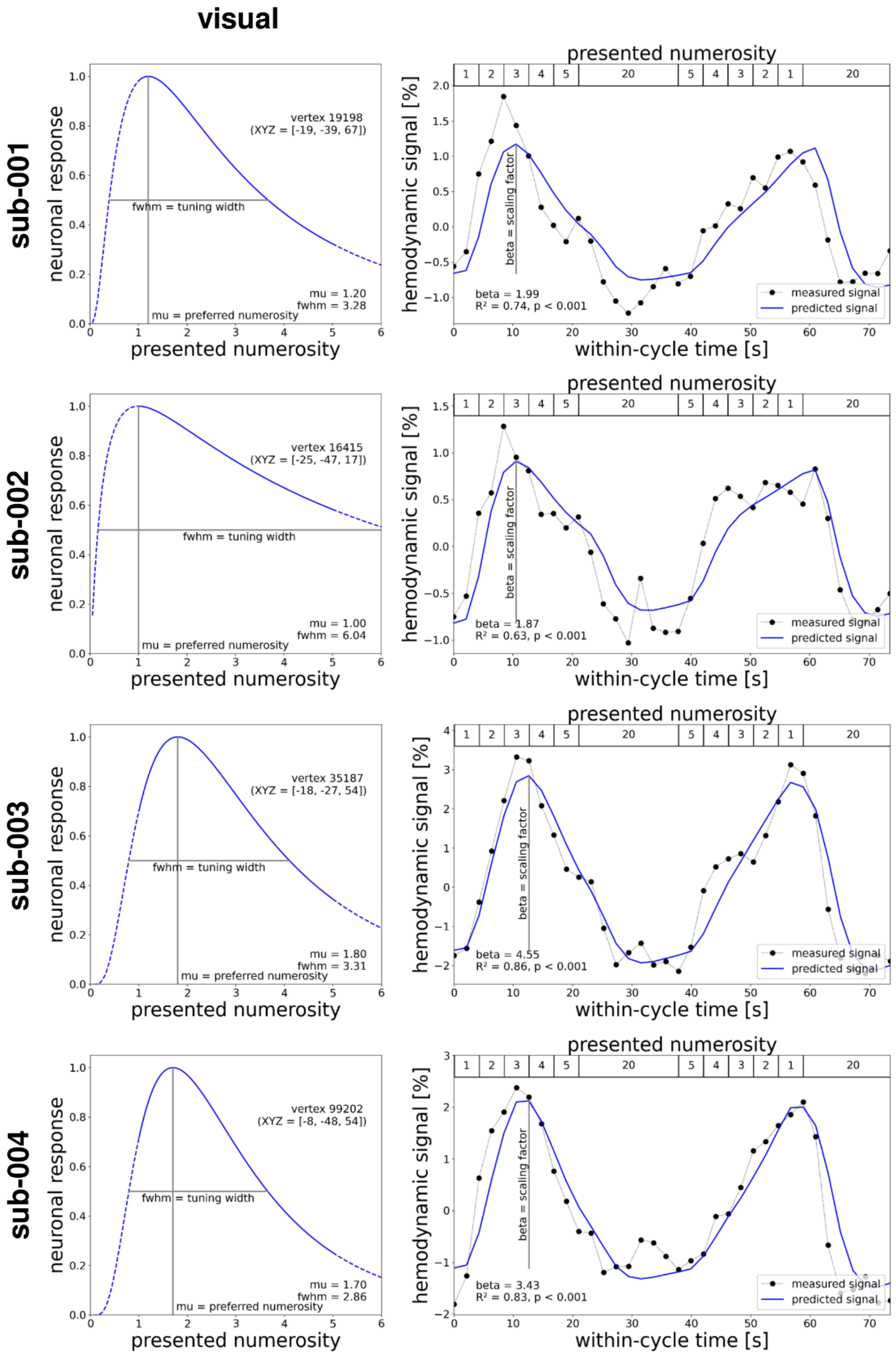

Figure S1: Neural tuning and hemodynamic responses to visual numerosity (page 1 of 3). Neural response functions (left) and hemodynamic responses measured using fMRI during visual numerosity presentation (right) were extracted from the vertex with the highest R^2.

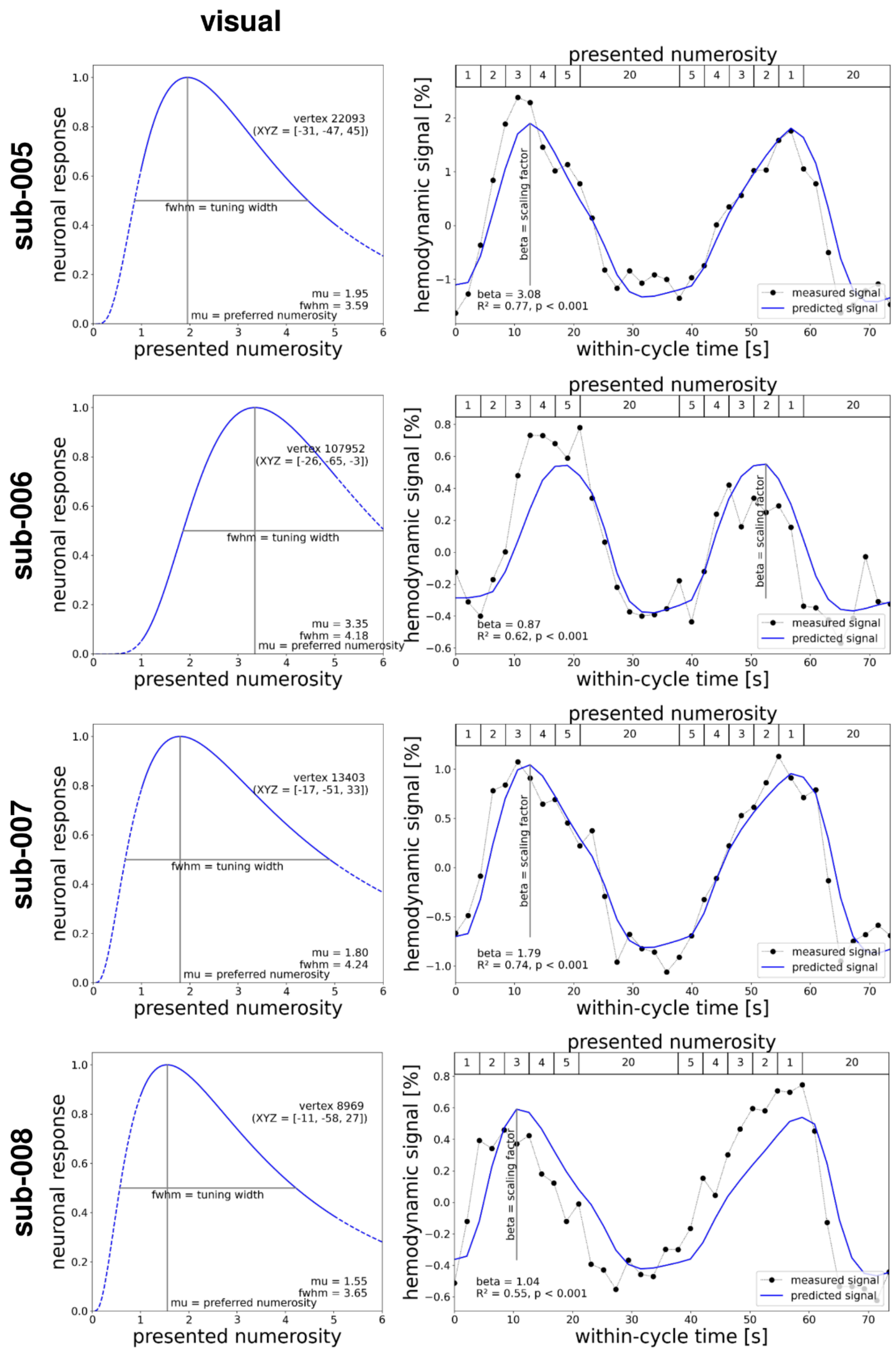

Figure S1: Neural tuning and hemodynamic responses to visual numerosity (page 2 of 3).

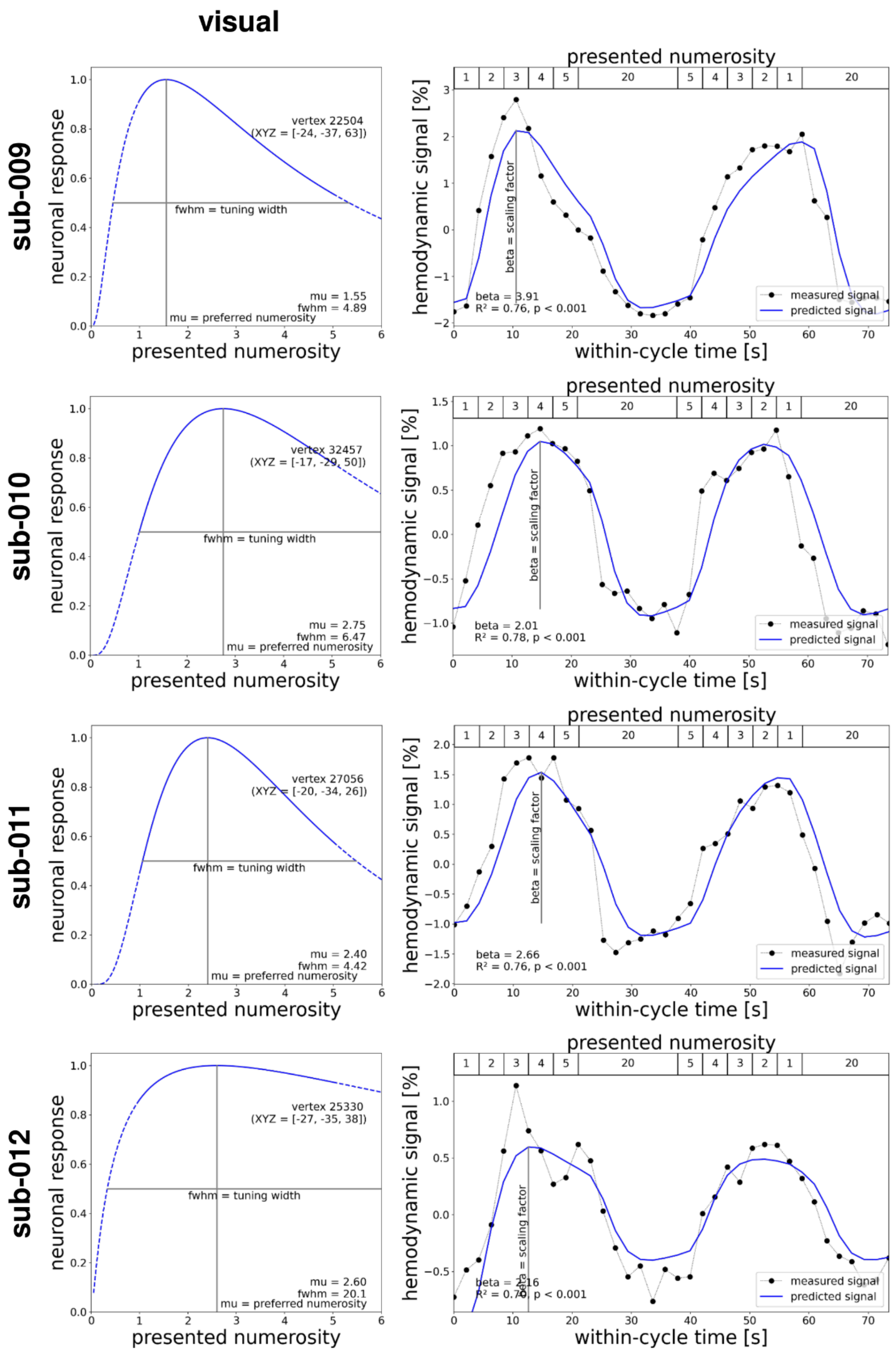

Figure S1: Neural tuning and hemodynamic responses to visual numerosity (page 3 of 3).

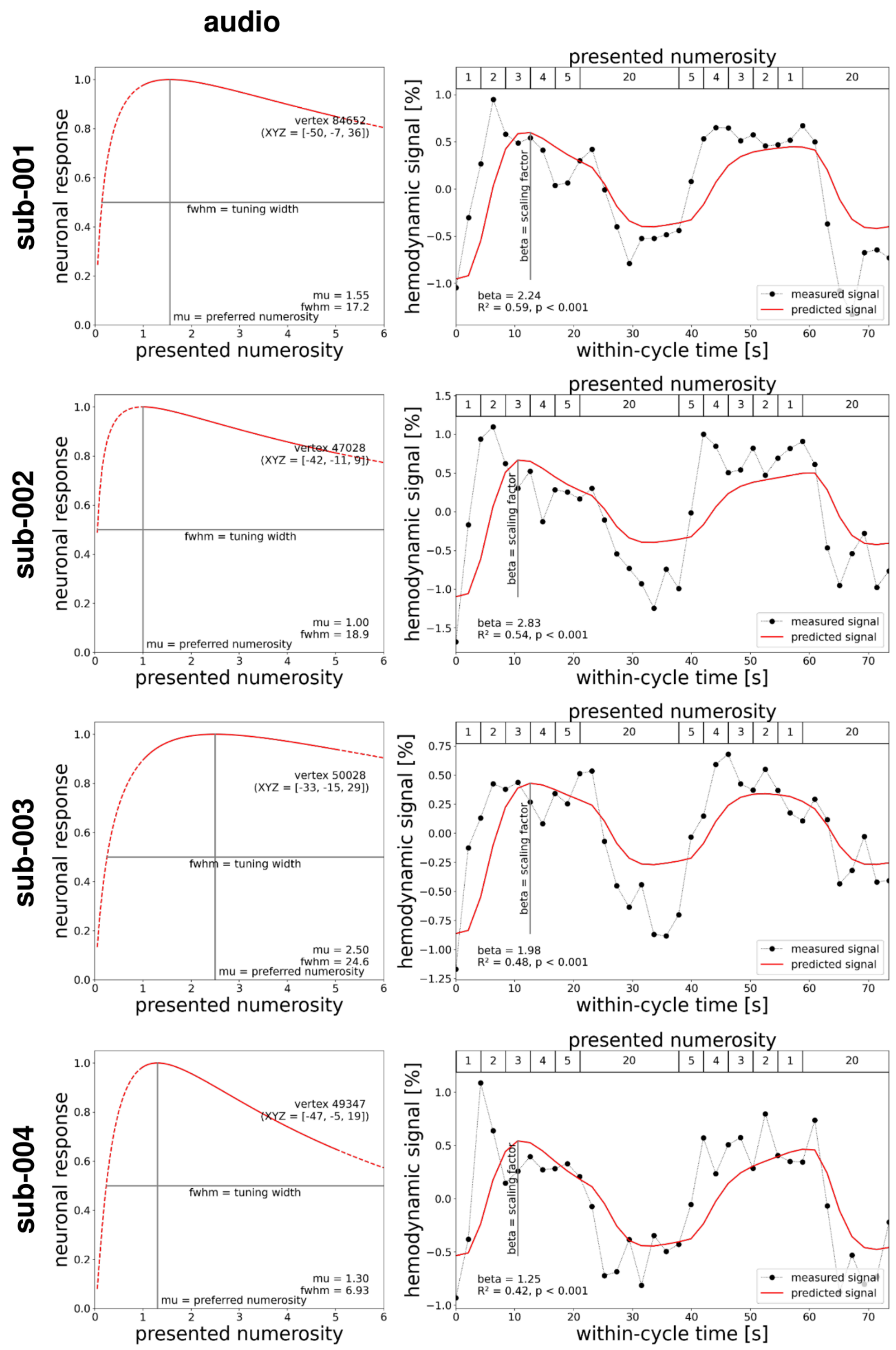

Figure S2: Neural tuning and hemodynamic responses to auditory numerosity (page 1 of 3). Neural response functions (left) and hemodynamic responses measured using fMRI during auditory numerosity presentation (right) were extracted from the vertex with the highest R^2.

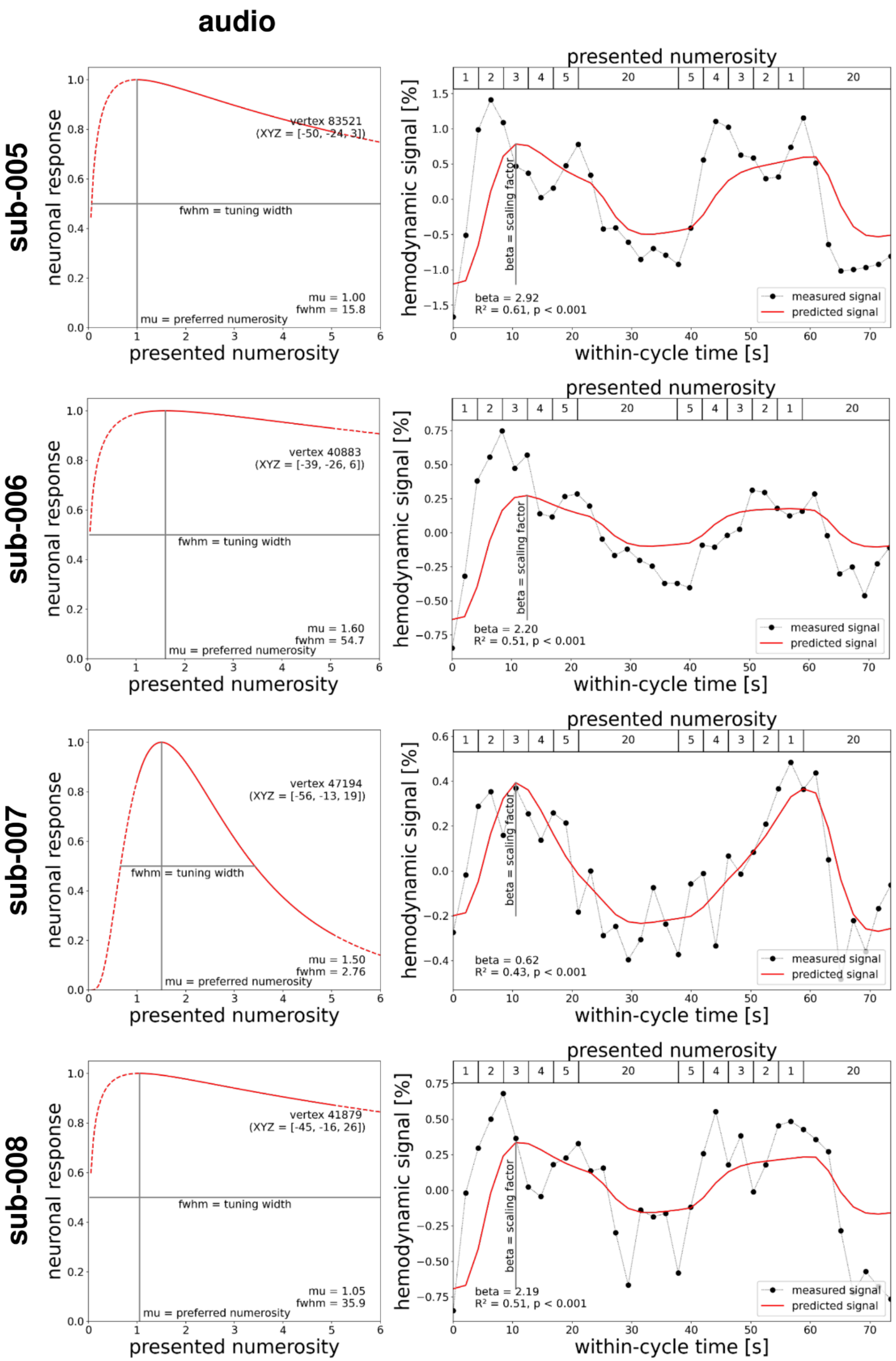

Figure S2: Neural tuning and hemodynamic responses to auditory numerosity (page 2 of 3).

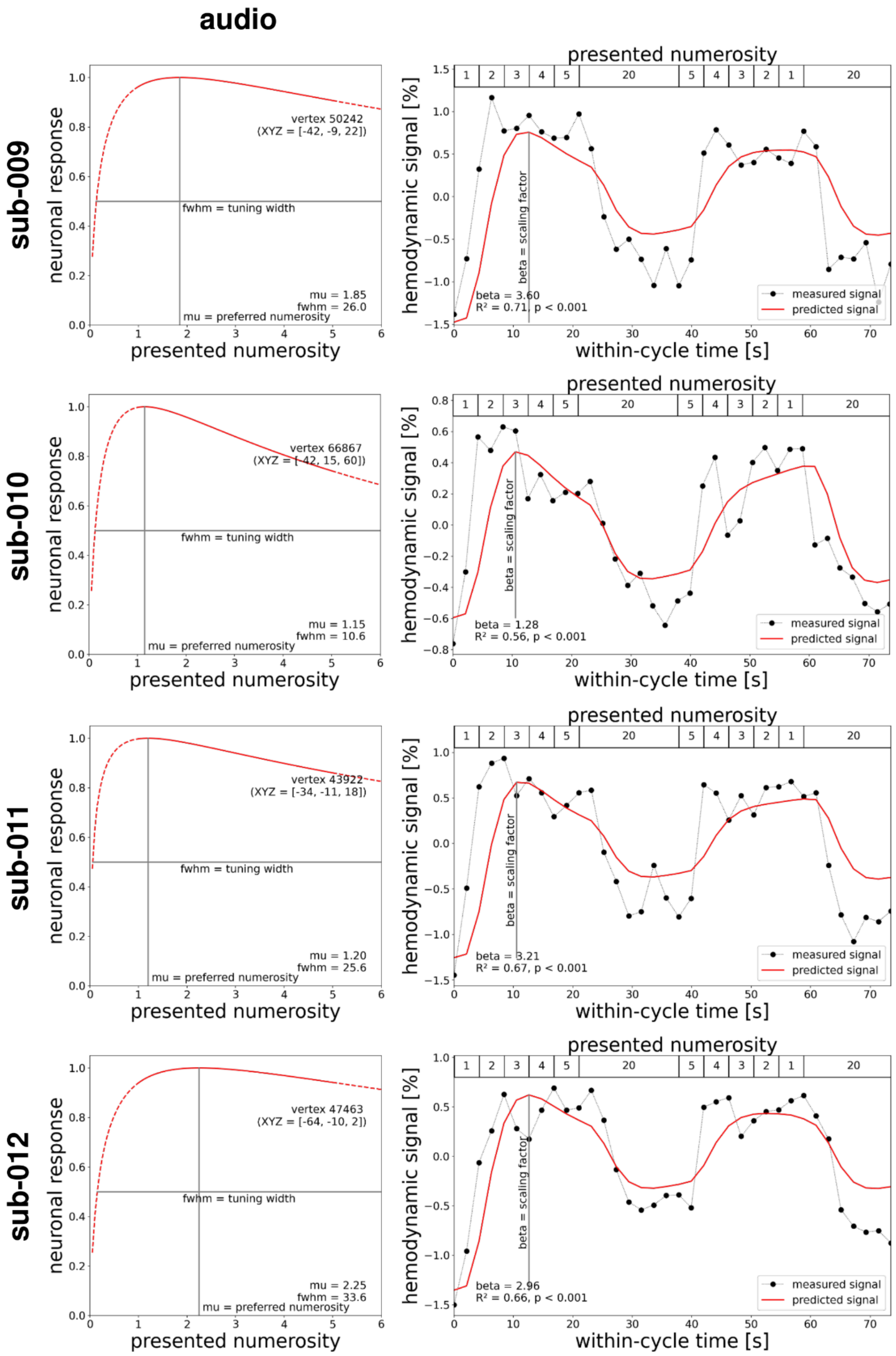

Figure S2: Neural tuning and hemodynamic responses to auditory numerosity (page 3 of 3).

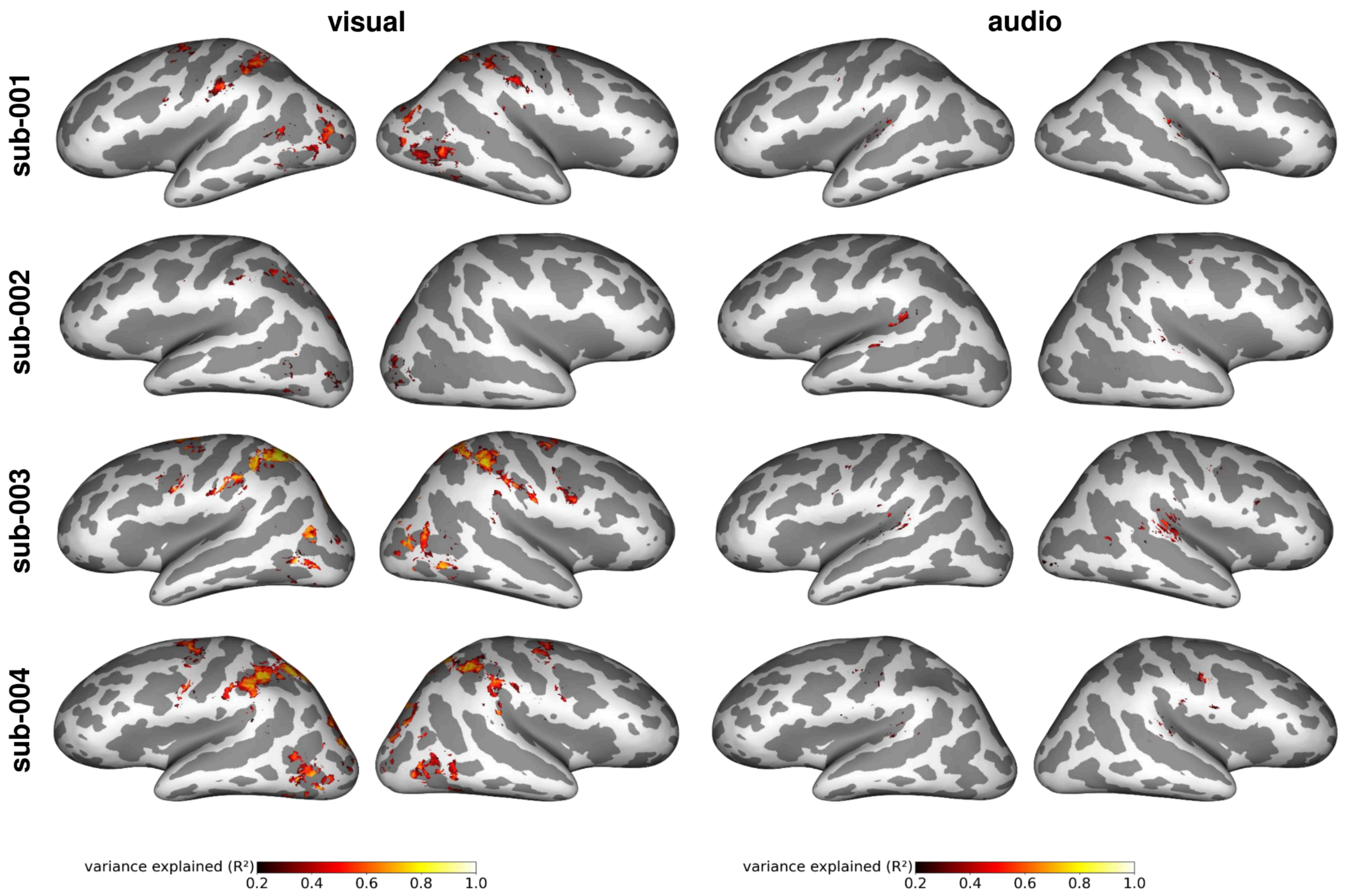

Figure S3: Variance explained by the neural tuning model (page 1 of 3). Inflated surface maps show R^2 of vertices in each subject's native space in which the neural tuning model explains at least 20% of the variance during visual and auditory numerosity perception.

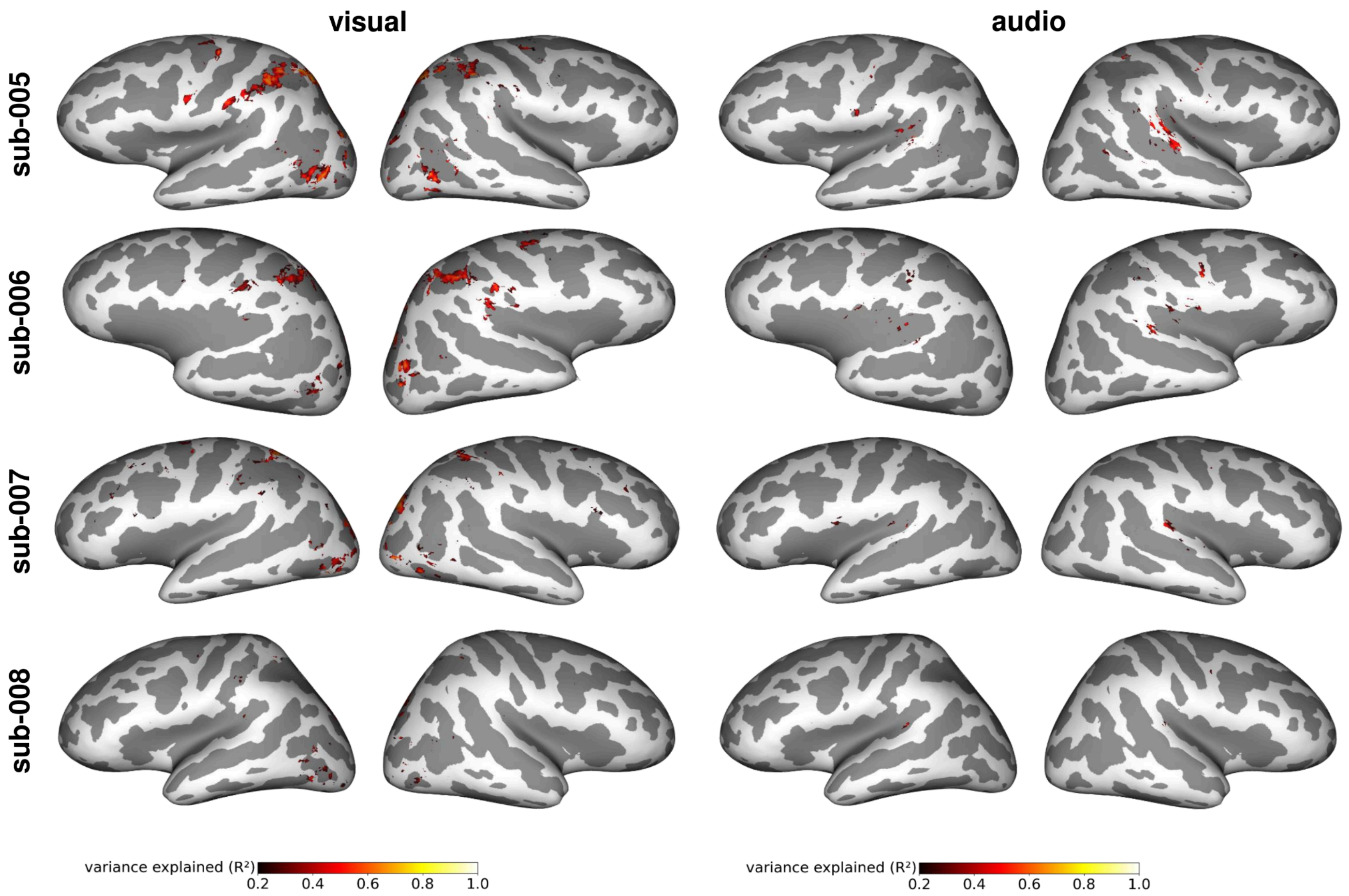

Figure S3: Variance explained by the neural tuning model (page 2 of 3).

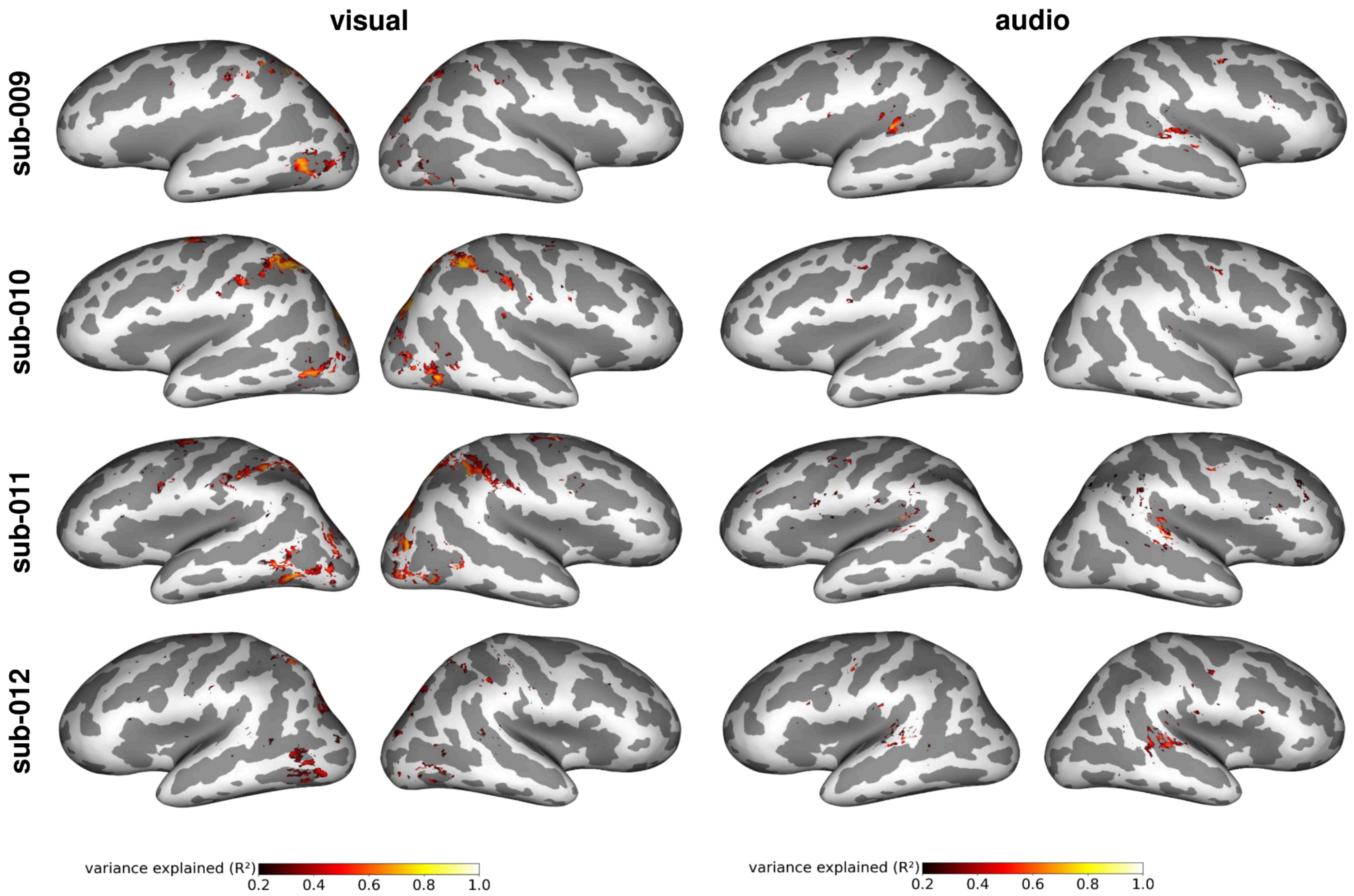

Figure S3: Variance explained by the neural tuning model (page 3 of 3).

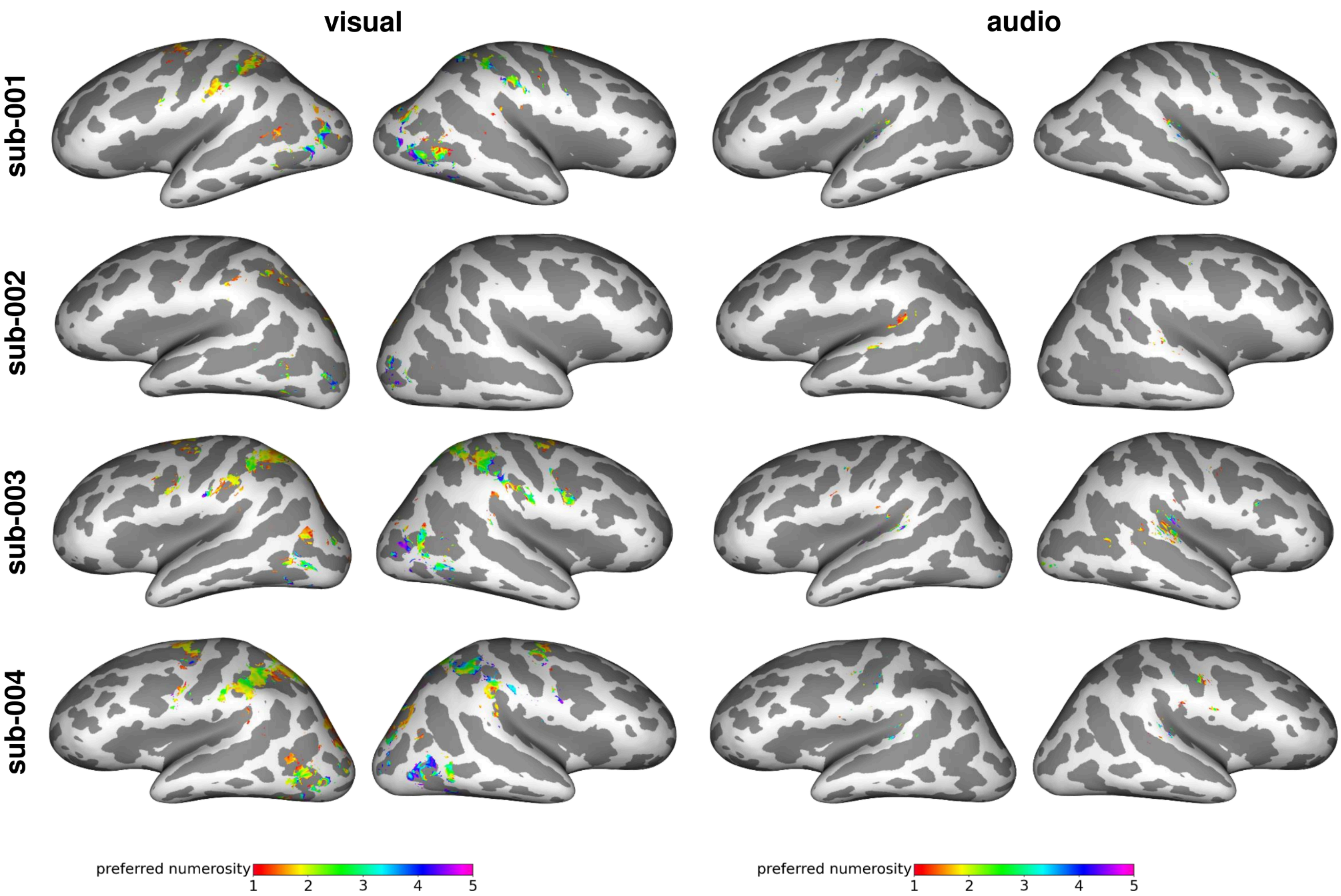

Figure S4: Preferred numerosity maps (page 1 of 3). Inflated surface maps show preferred numerosity of vertices in each subject's native space in which the neural tuning model explains at least 20% of the variance during visual and auditory numerosity perception.

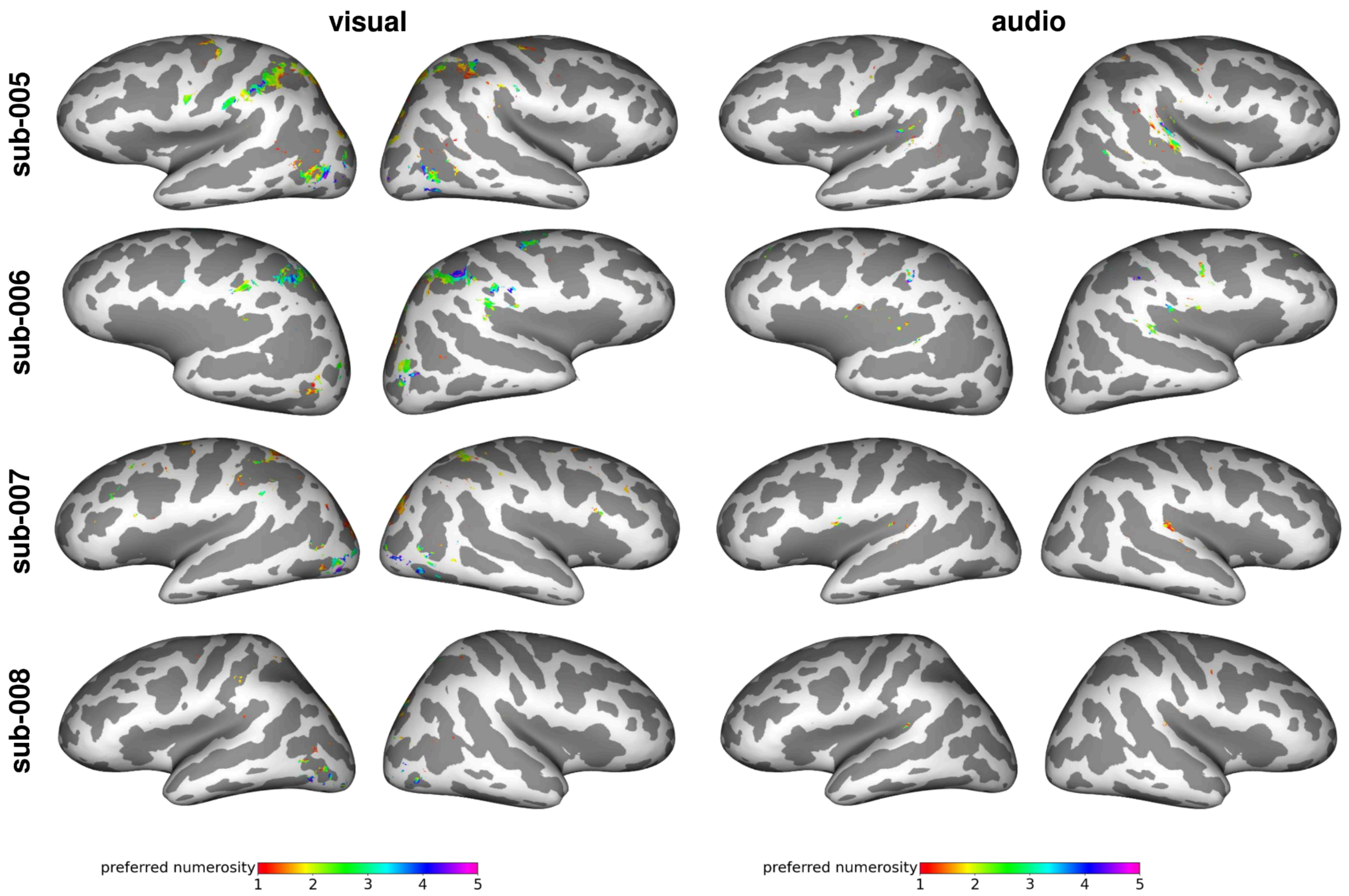

Figure S4: Preferred numerosity maps (page 2 of 3).

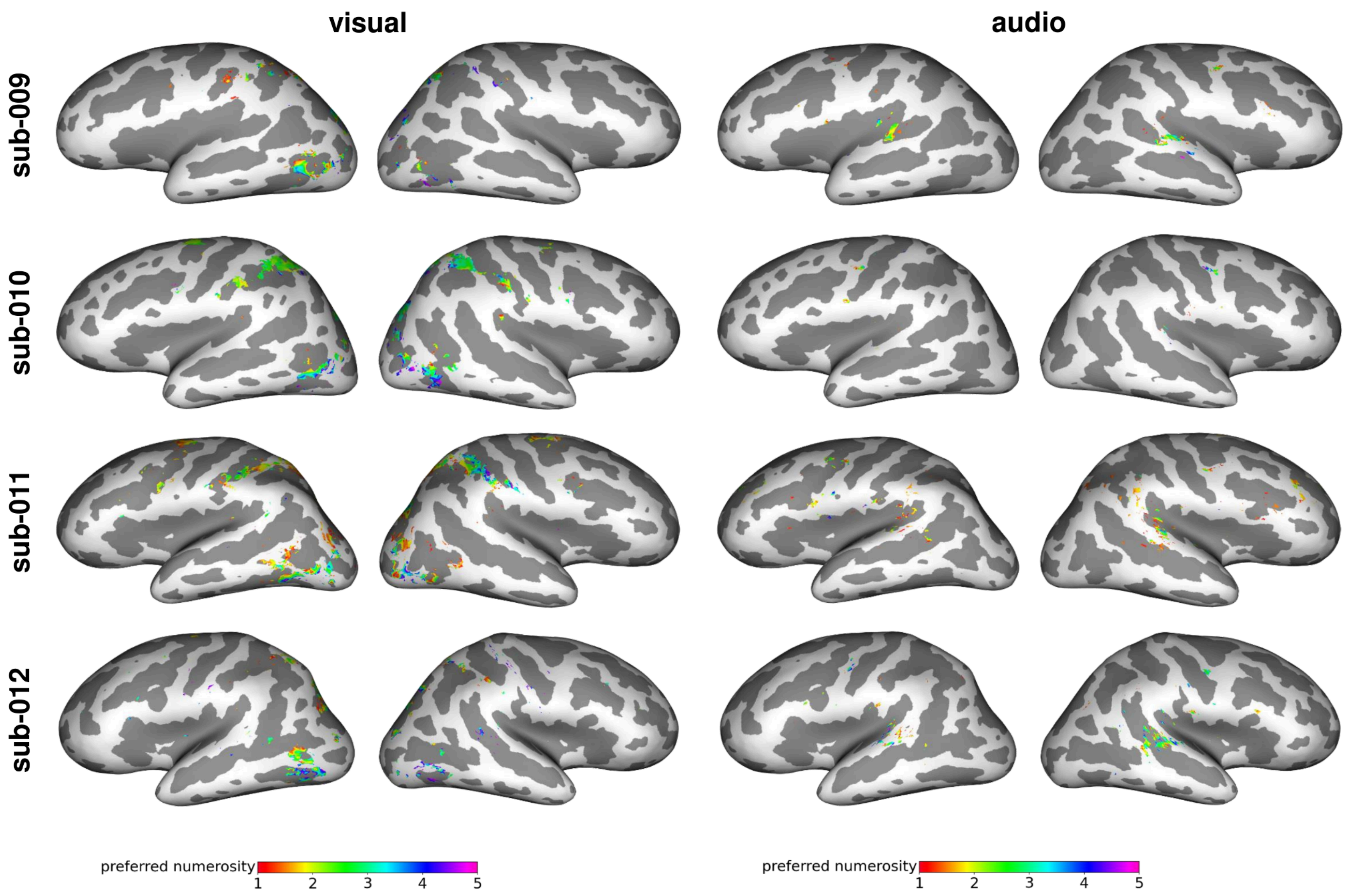

Figure S4: Preferred numerosity maps (page 3 of 3).

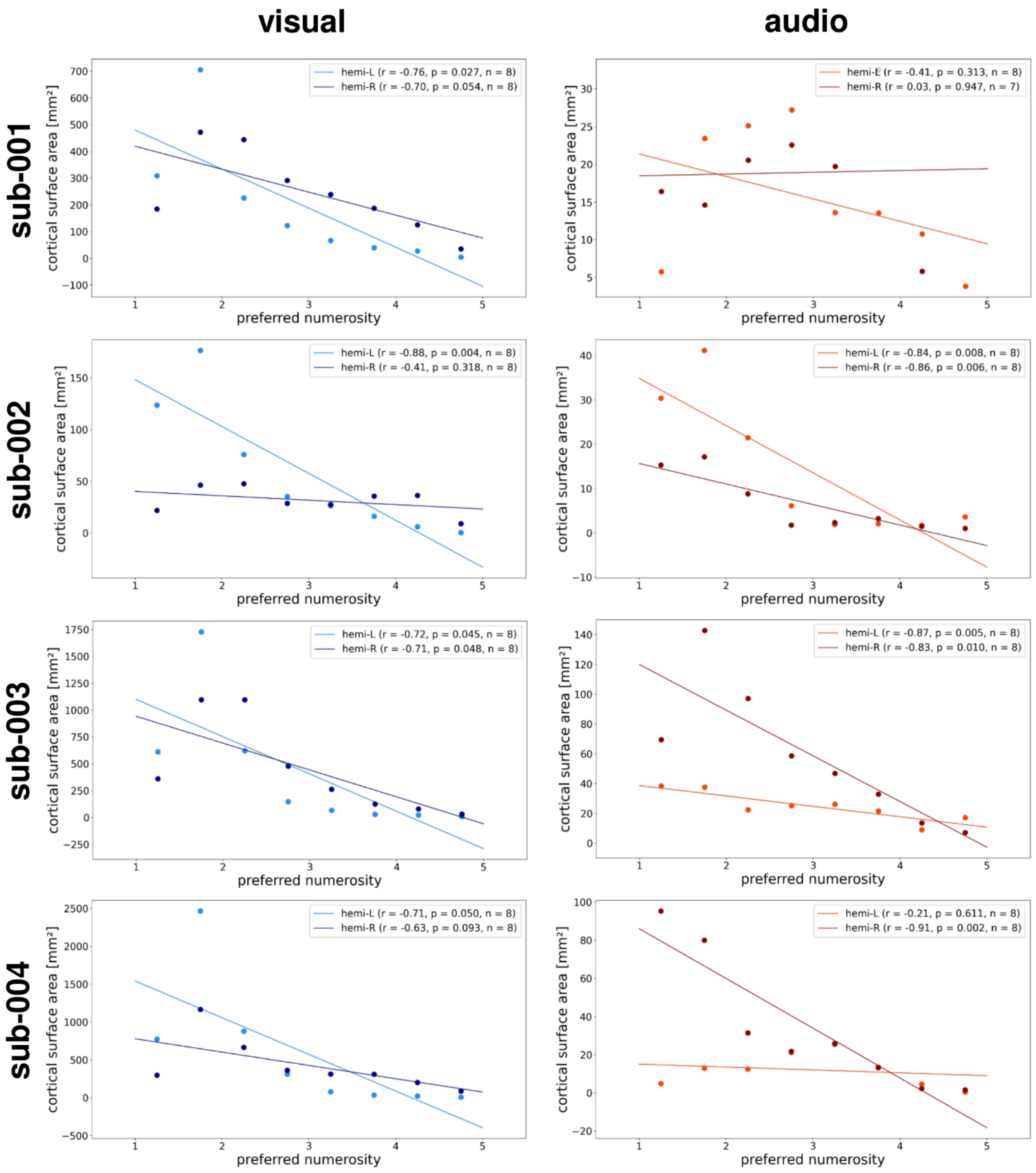

Figure S5: Linear associations between preferred numerosity and surface area (page 1 of 3). Cortical surface area decreases as a function of preferred numerosity in each subject perceiving either visual (left) or auditory (right) numerosity.

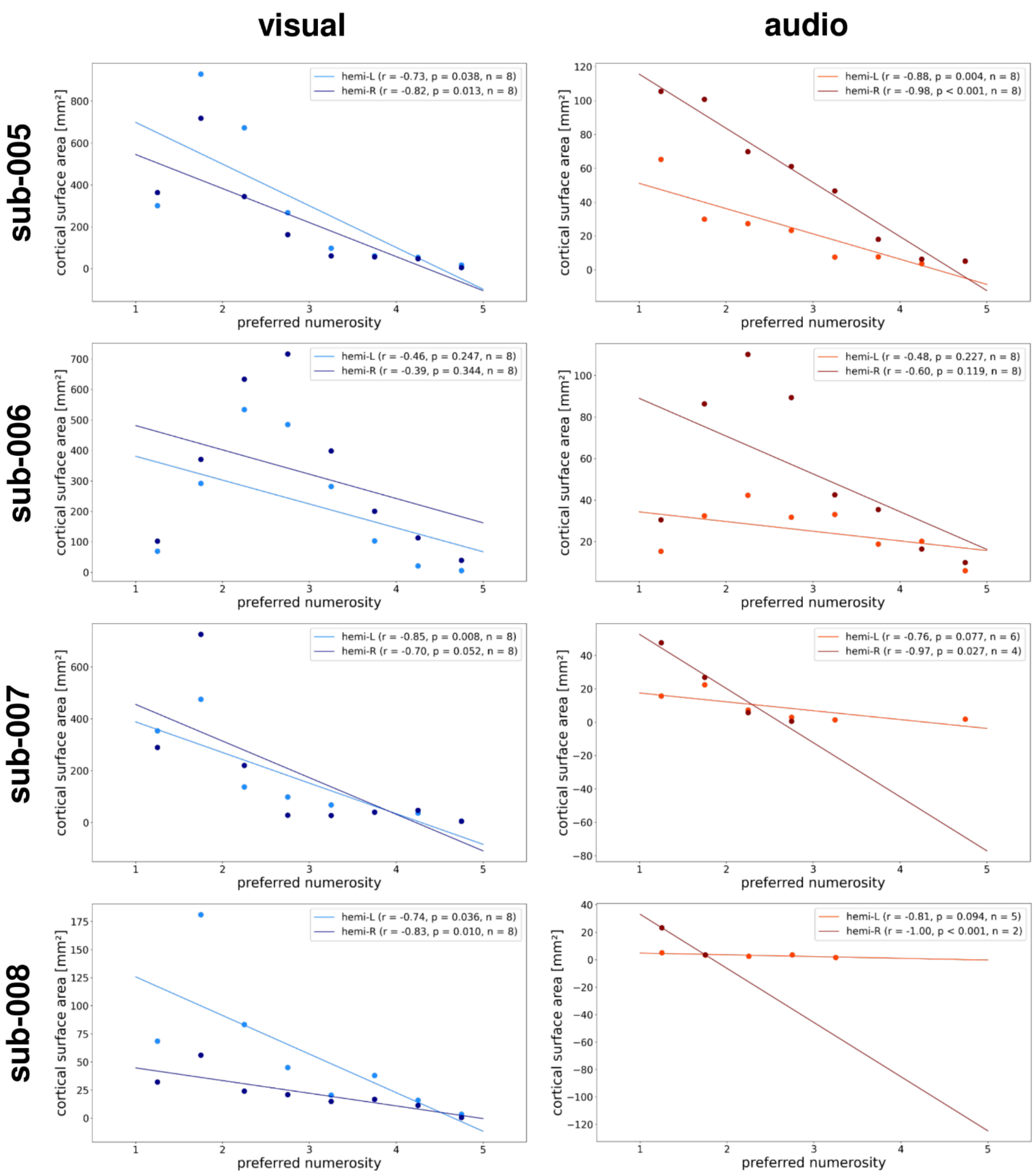

Figure S5: Linear associations between preferred numerosity and surface area (page 2 of 3).

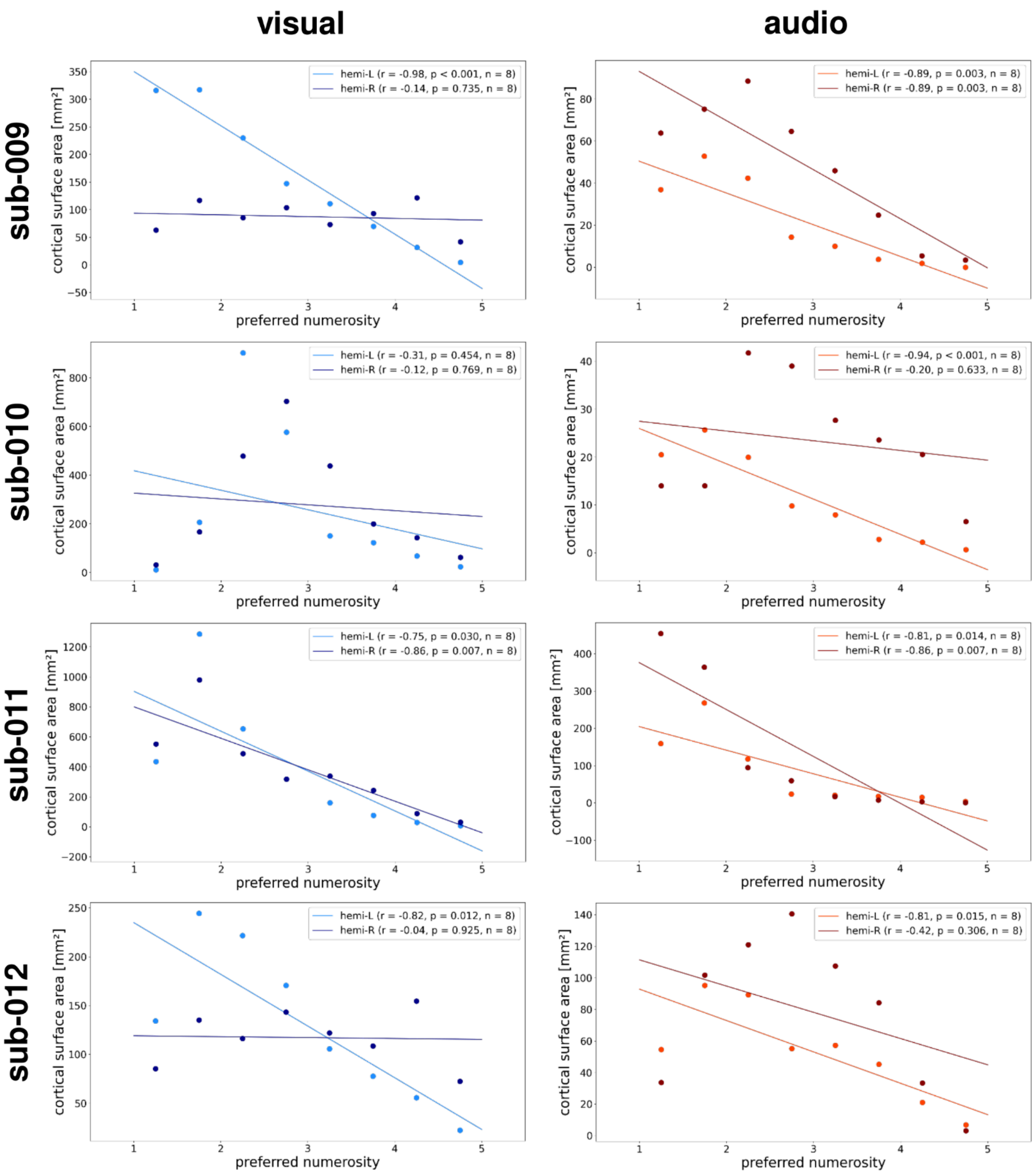

Figure S5: Linear associations between preferred numerosity and surface area (page 3 of 3).

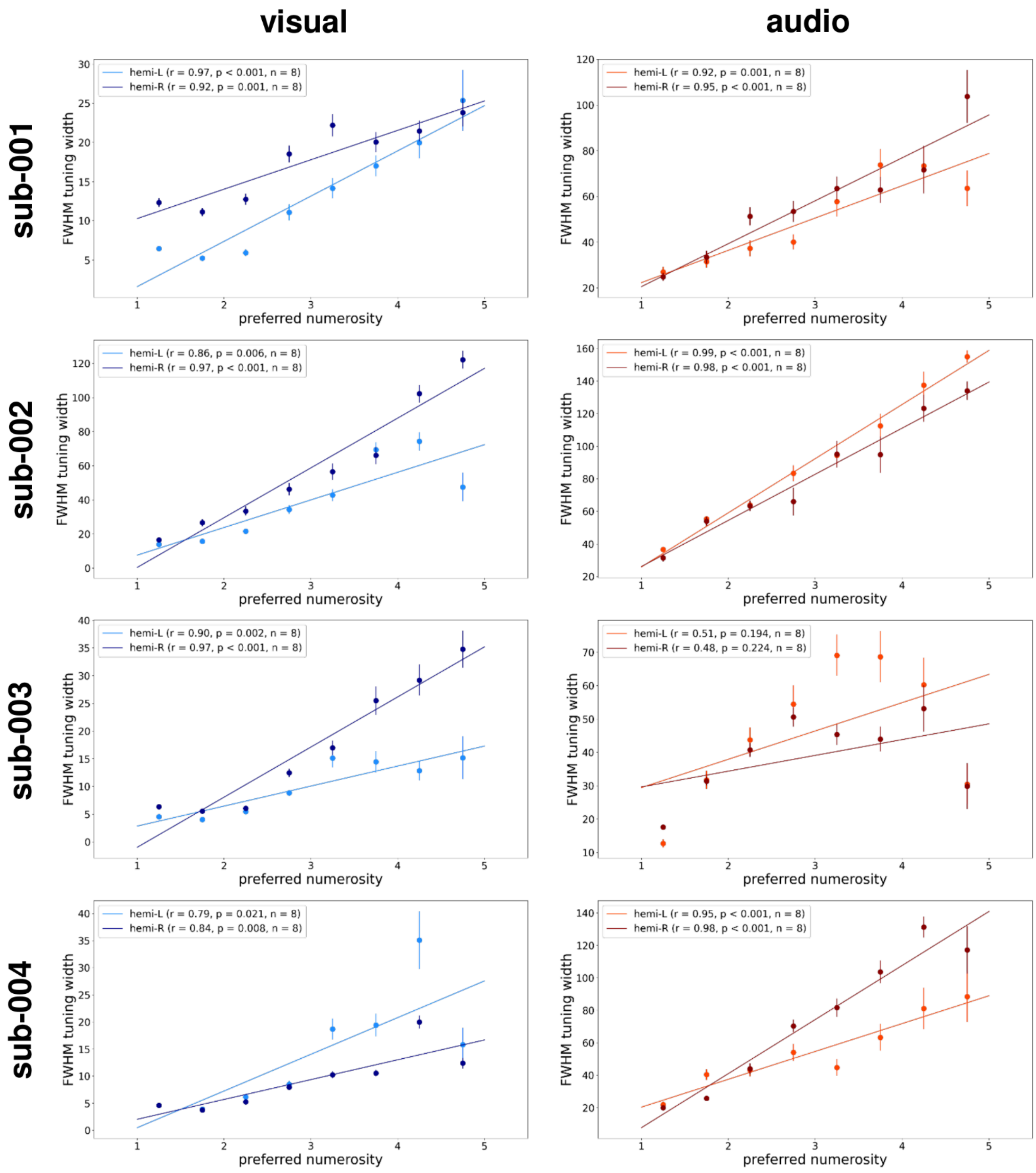

Figure S6: Linear associations between preferred numerosity and tuning width (page 1 of 3). Full width at half maximum (FWHM) increases as a function of preferred numerosity in each subject perceiving either visual (left) or auditory (right) numerosity.

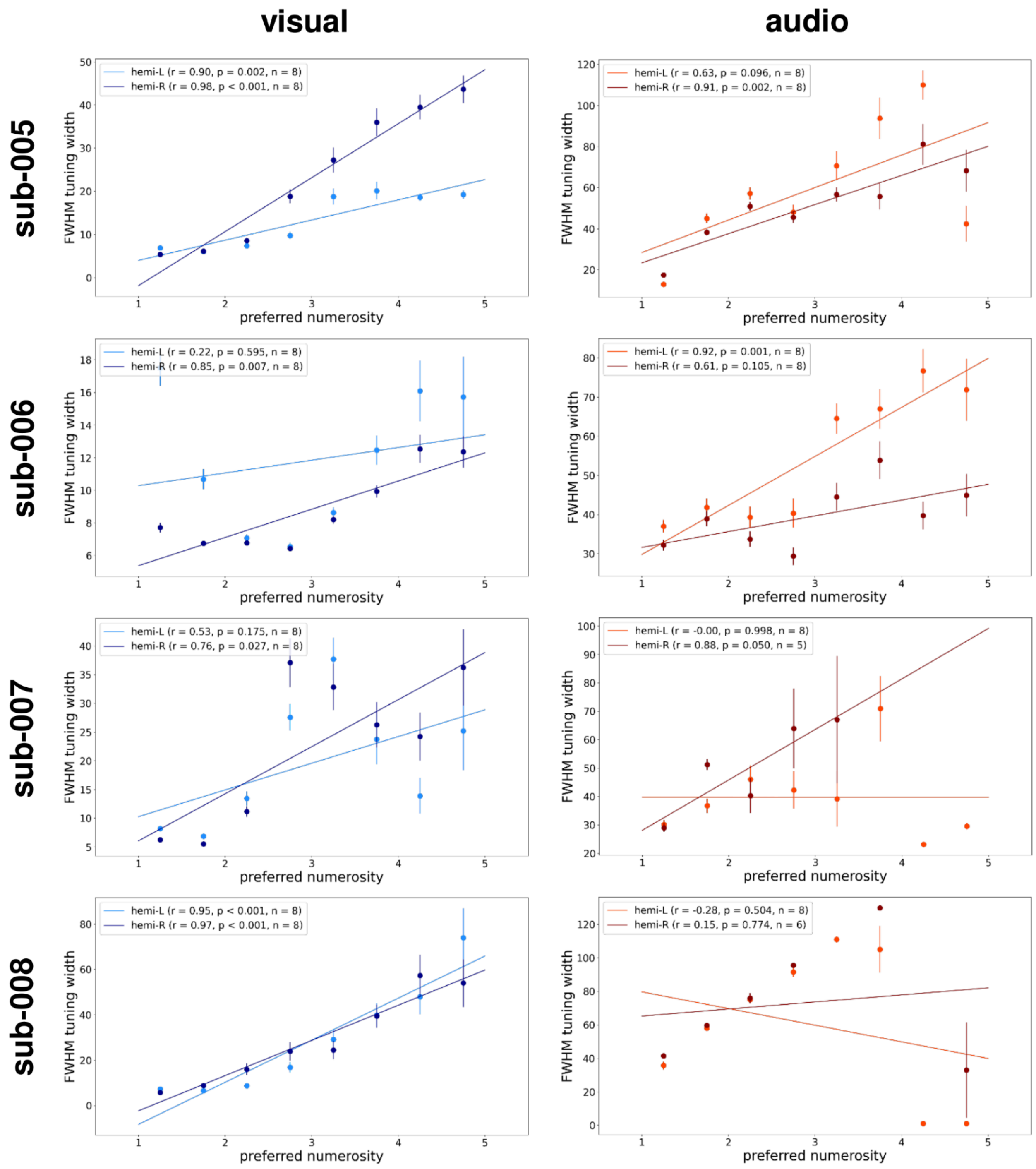

Figure S6: Linear associations between preferred numerosity and tuning width (page 2 of 3).

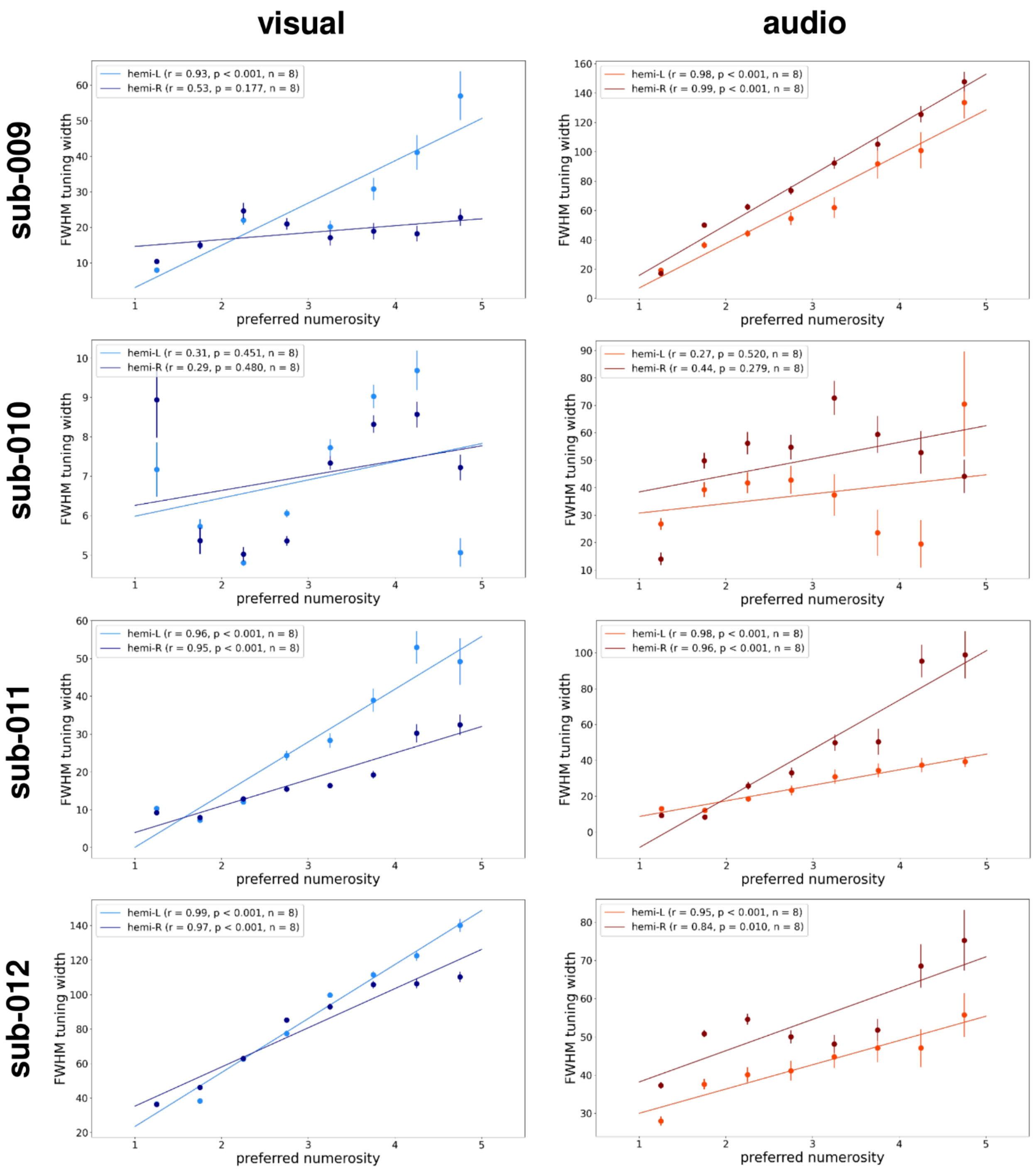

Figure S6: Linear associations between preferred numerosity and tuning width (page 3 of 3).

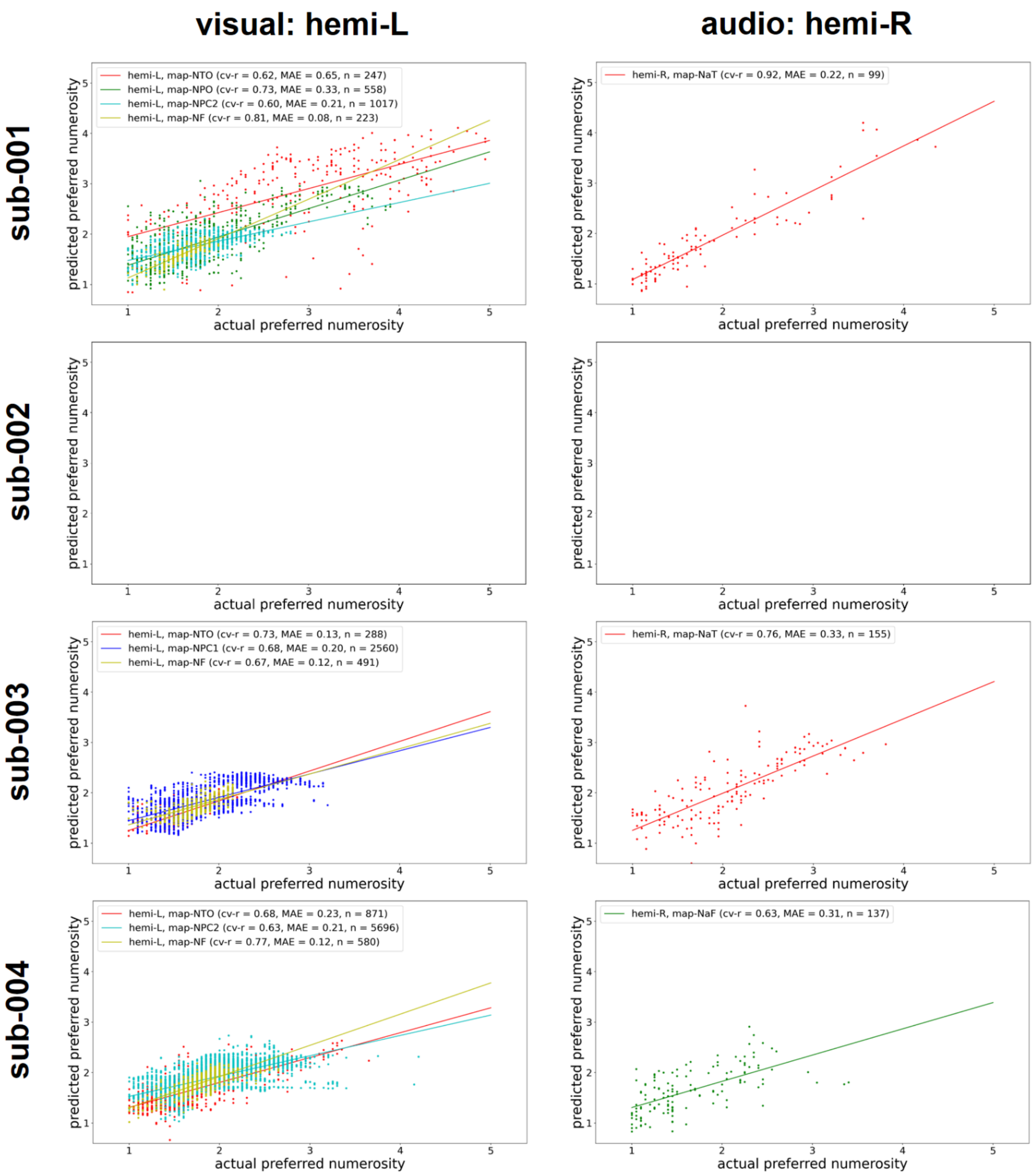

Figure S7: Fitted and preferred numerosity of numerotopic maps (page 1 of 3). Preferred numerosity can be predicted from surface coordinates, indicating a progression of preferred numerosity on the cortical surface within visual and auditory numerotopic maps.

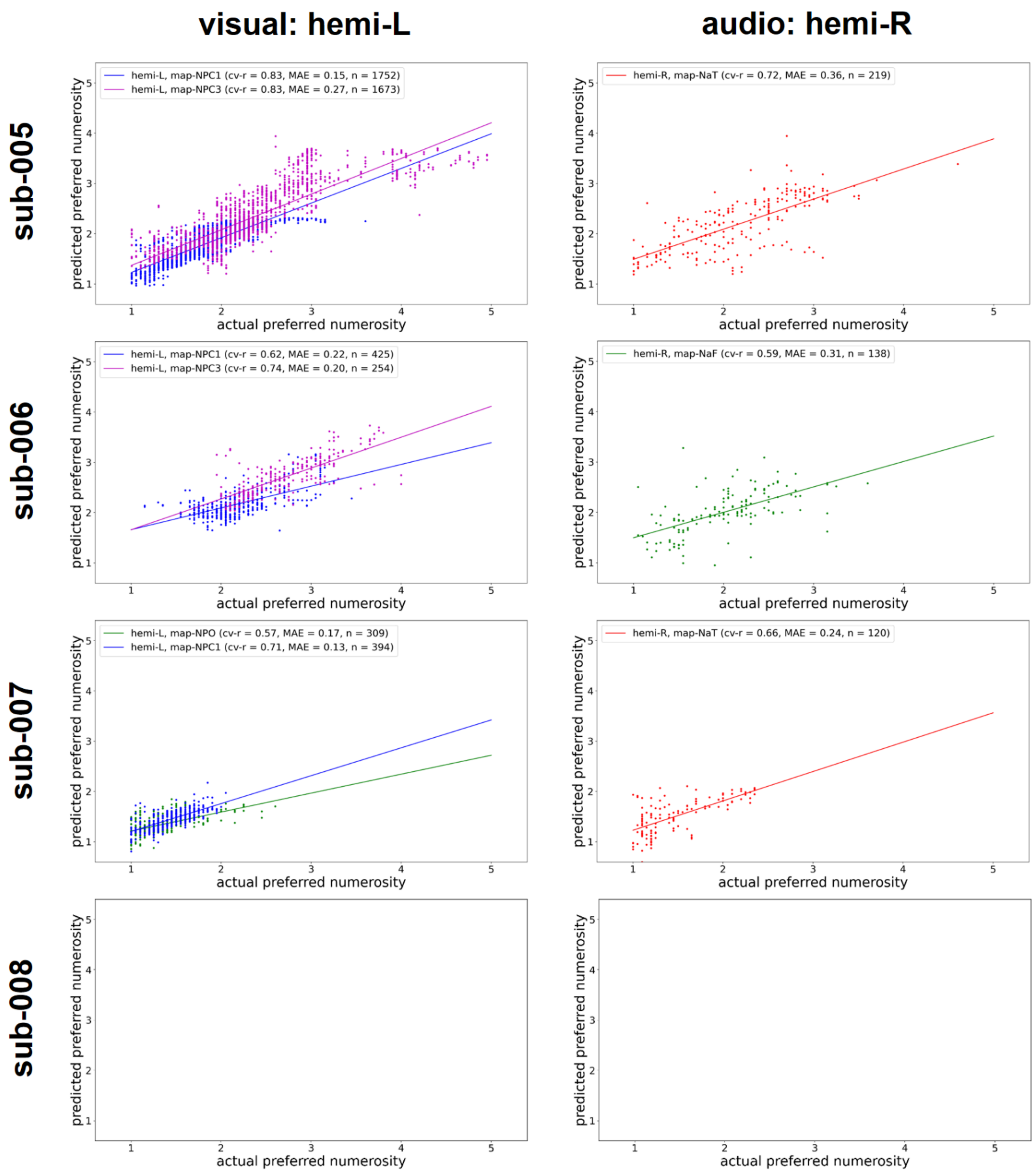

Figure S7: Fitted and preferred numerosity of numerotopic maps (page 2 of 3).

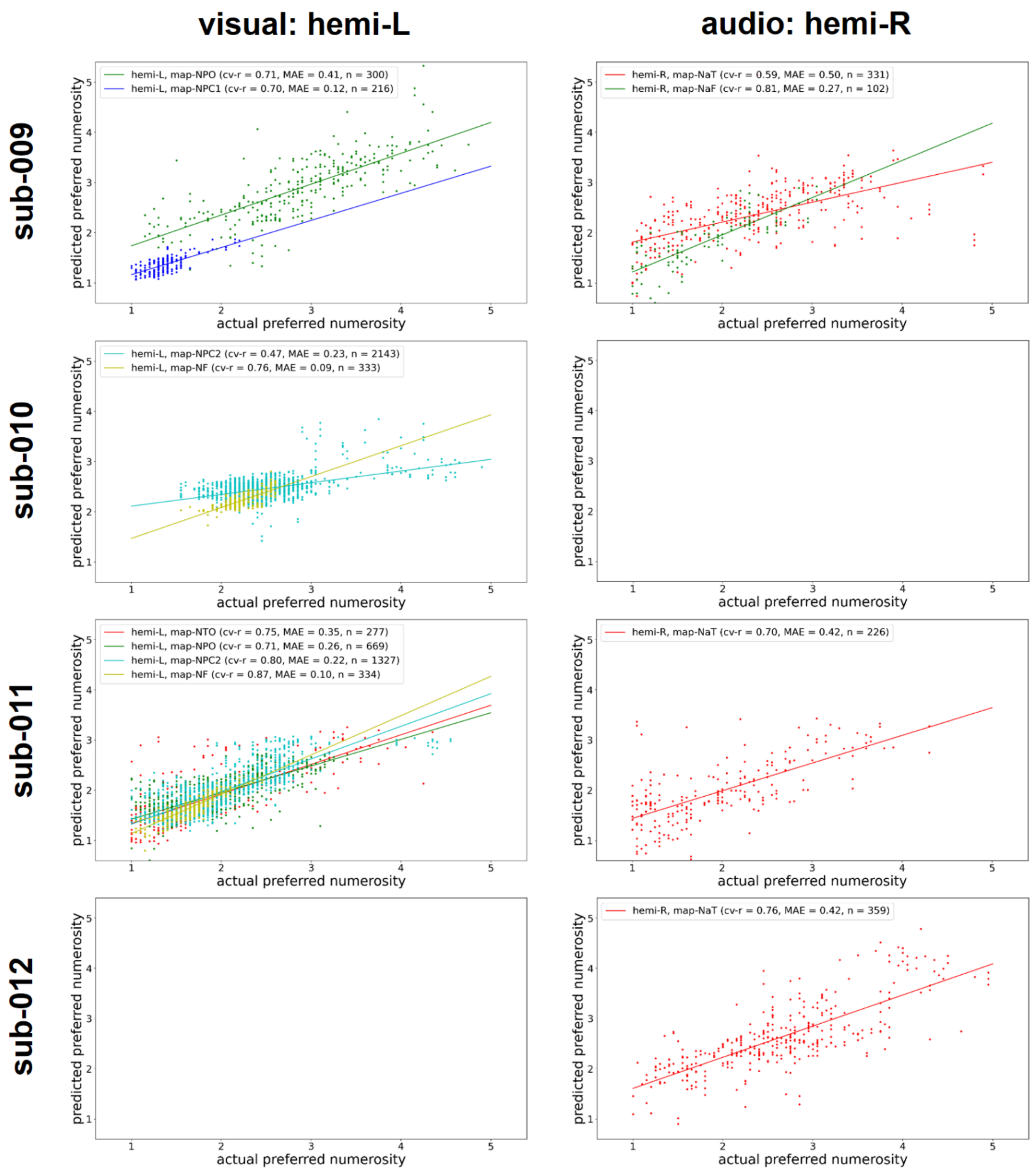

Figure S7: Fitted and preferred numerosity of numerotopic maps (page 3 of 3).

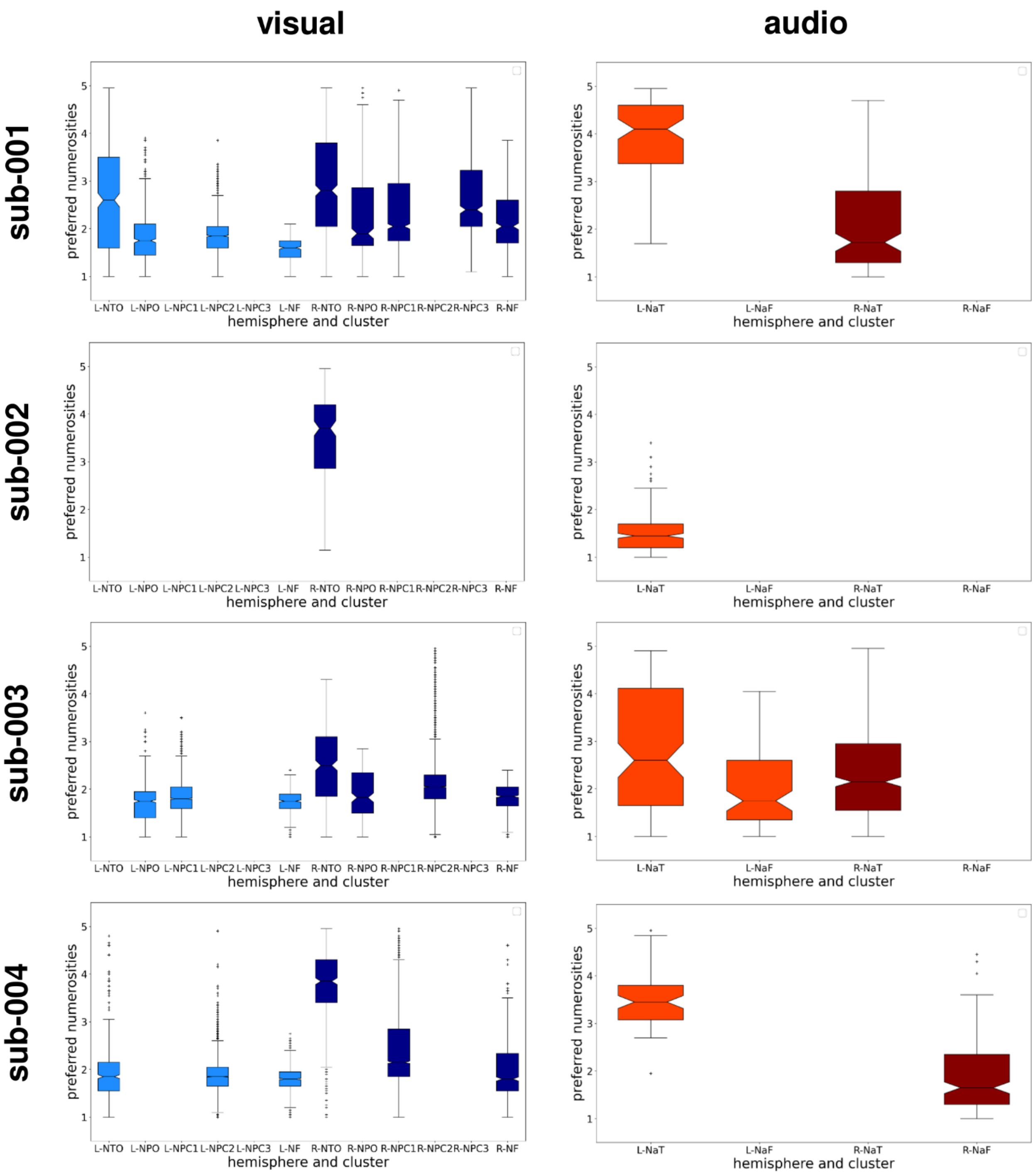

Figure S8: Range of preferred numerosities within numerotopic maps (page 1 of 3). Preferred numerosities of all supra-threshold vertices within up to six visual and up to two auditory numerosity maps per hemisphere are shown as boxplots. Horizontal lines within boxes mark the median. Whiskers indicate the most extreme points that are still within 1.5 x IQR from the upper or lower quartile. No data are shown in accordance with predefined thresholds whenever clusters did not exceed a surface area of 50 mm^2 or when the minimum distance of a cluster from prespecified numerosity maps was larger than 25 mm.

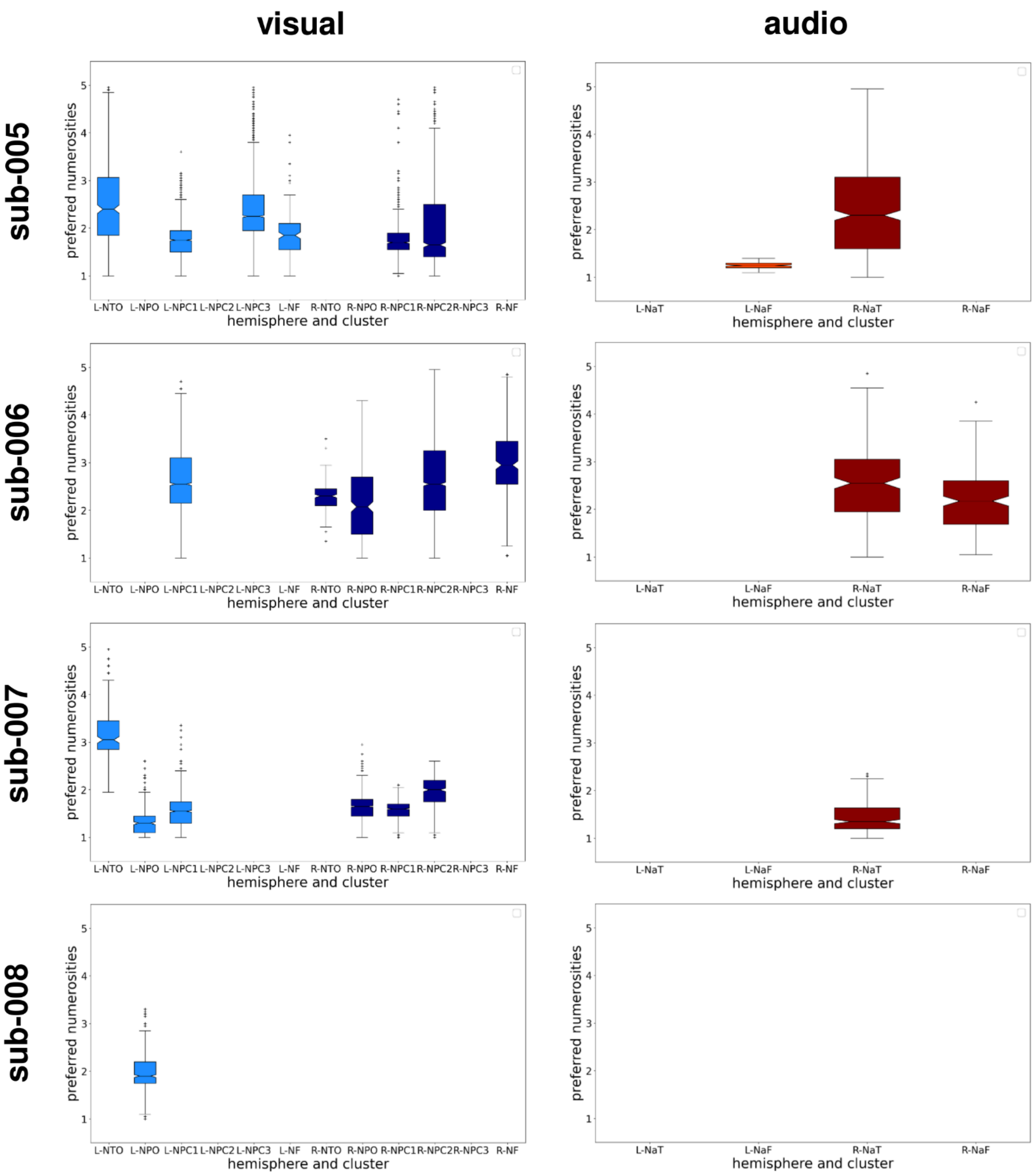

Figure S8: Range of preferred numerosities within numerotopic maps (page 2 of 3).

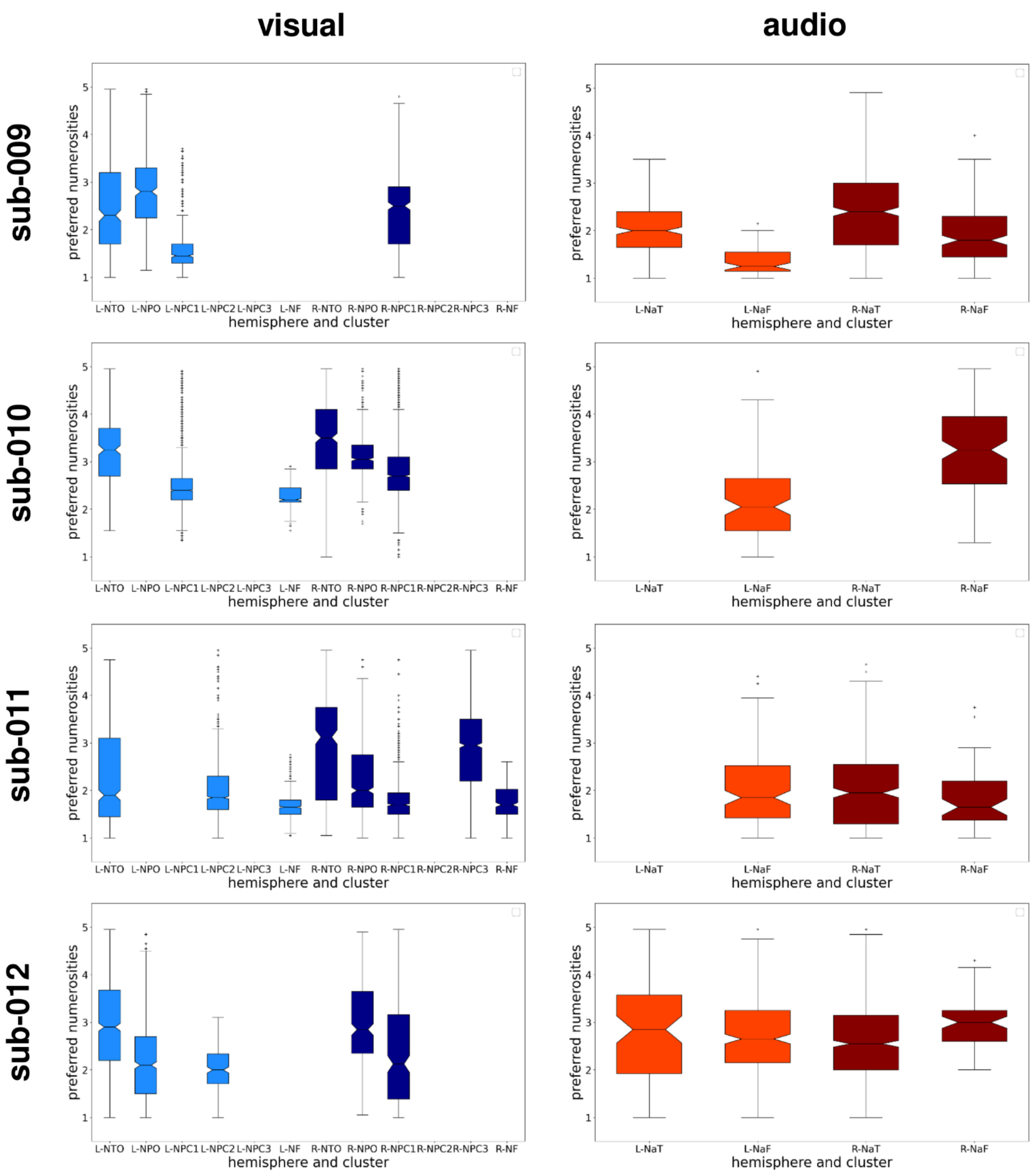

Figure S8: Range of preferred numerosities within numerotopic maps (page 3 of 3).

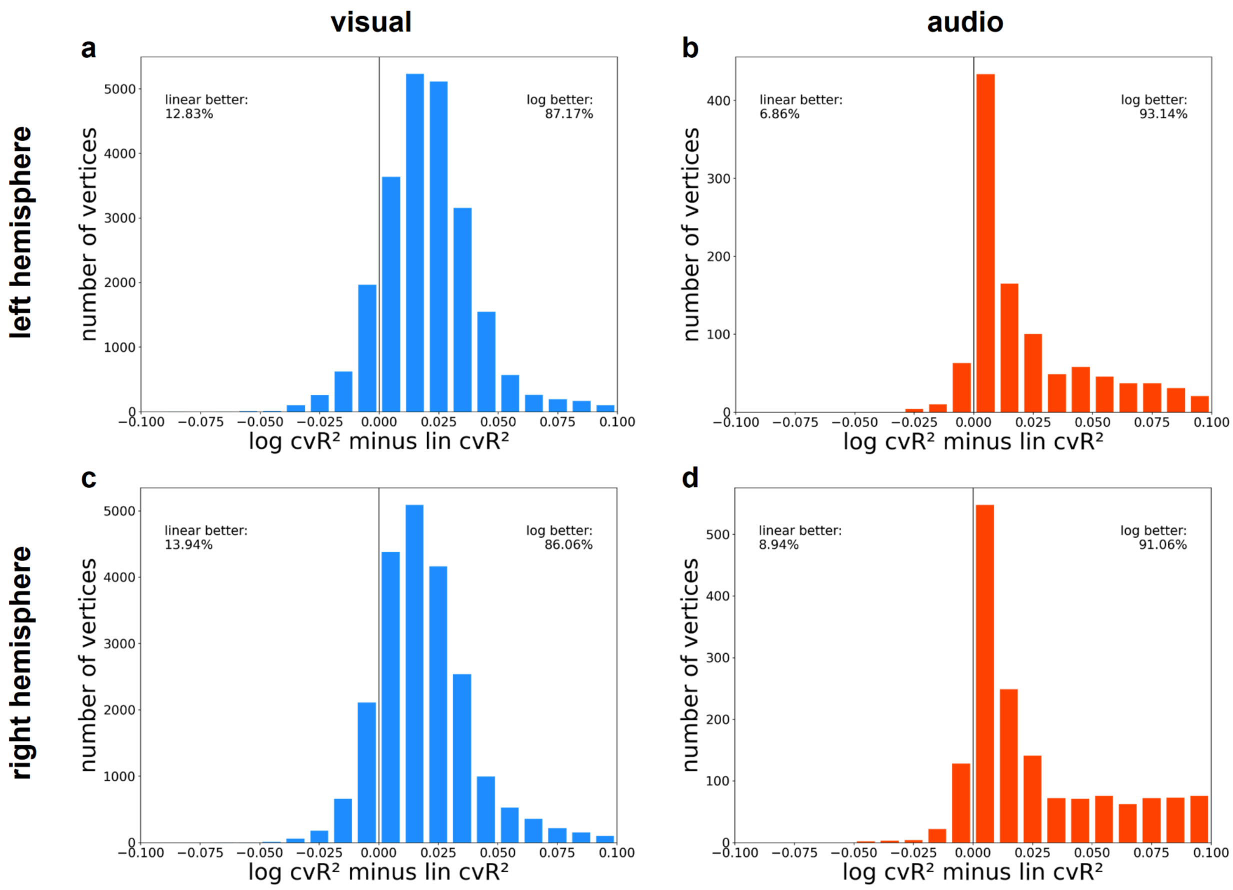

Figure S9: Comparison of linear and logarithmic tuning functions. Panels display differences of cross-validated coefficients of determination (cvR²) between linear and logarithmic tuning function models estimated from signals **a,b** in vertices in the left hemisphere and **c,d** vertices in the right hemisphere for **a,c** visual numerosity experiments and **b,d** auditory numerosity experiments. Whenever this difference is positive, logarithmic tuning models outperform linear tuning models. This figure follows Figure S5 in Harvey et al. (2013) and Figure 1E in Harvey et al. (2015). Comparisons include all vertices in which the explained variance of both, linear and logarithmic models, was significantly larger than zero ($p < 0.05$, Bonferroni-corrected for number of vertices in subject and hemisphere).

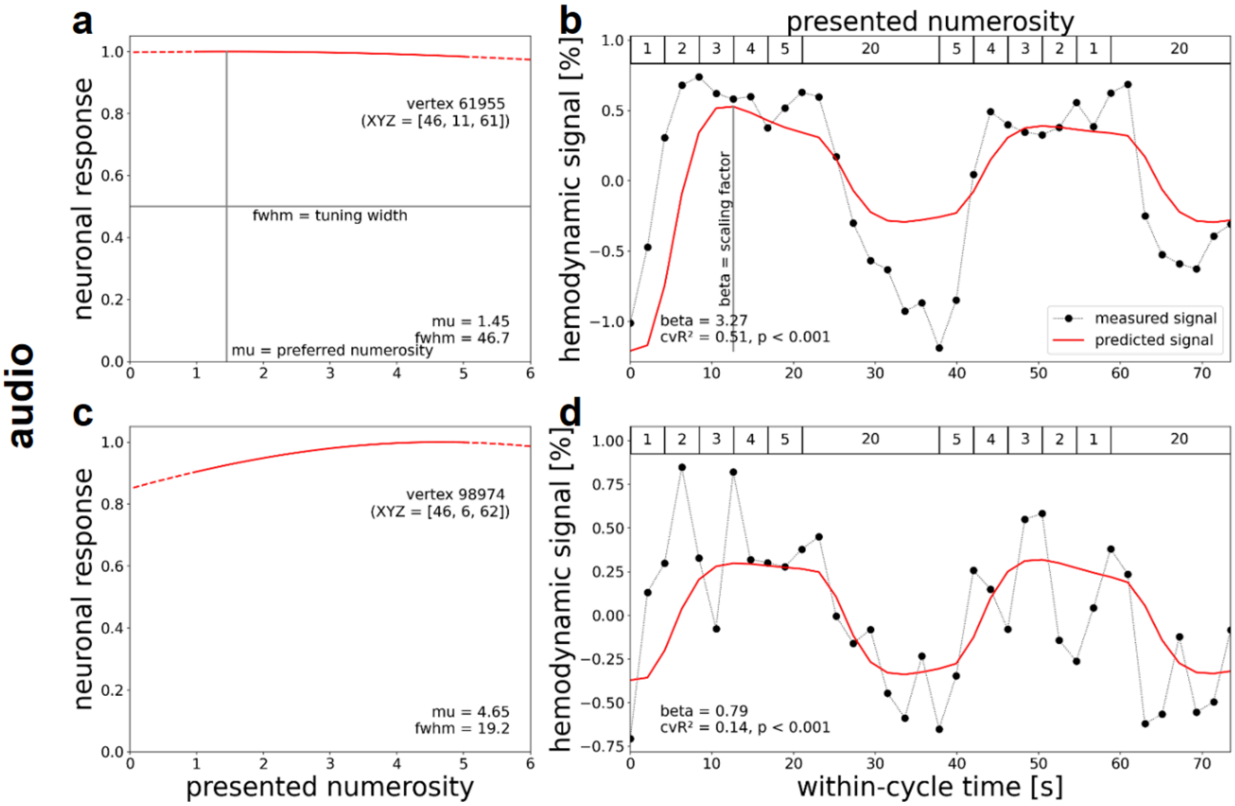

Figure S10: Linear tuning function model for auditory numerosity. The layout of this figure follows that of Fig. 2e–h, except that tuning functions describing hemodynamic responses were extracted using a linear tuning model. **a,c** Linear Gaussian tuning functions are shown for **a** a small-numerosity vertex and **c** a larger-numerosity vertex per subject (XYZ = coordinates in FreeSurfer's fsnative space). These functions are described by a preferred numerosity (mu) and the full width at half maximum (fwhm) in linear numerosity space. **b,d** Combining neuronal tuning models with a hemodynamic forward model generated predicted time courses (solid lines) for auditory numerosity presentation. Comparing predicted to measured time courses (dotted lines, averaged across runs and cycles) yielded the cross-validated coefficient of determination (cvR^2) quantifying the out-of-sample variance explained by the model (calculated across averaged runs).

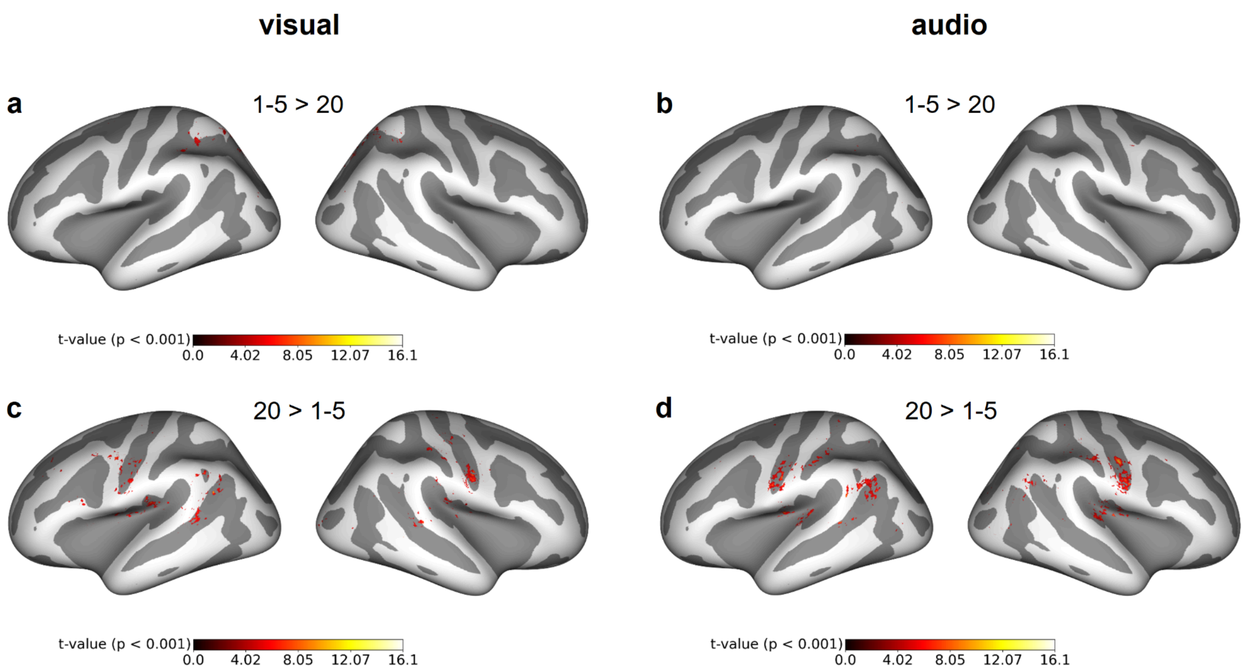

Figure S11: General Linear Model results. Panels display thresholded statistical parametric maps ($p < 0.001$, uncorrected) corresponding to **a,b** significant positive effects of small numerosity (1-5 > 20) and **c,d** significant negative effects of small numerosity (20 > 1-5) for **a,c** visual numerosity experiments and **b,d** auditory numerosity experiments. The first colorbar tick label ($t = 4.02$) corresponds to the critical t-value for a one-sample t-test with sample size $n = 12$ and significance level $\alpha = 0.001$.

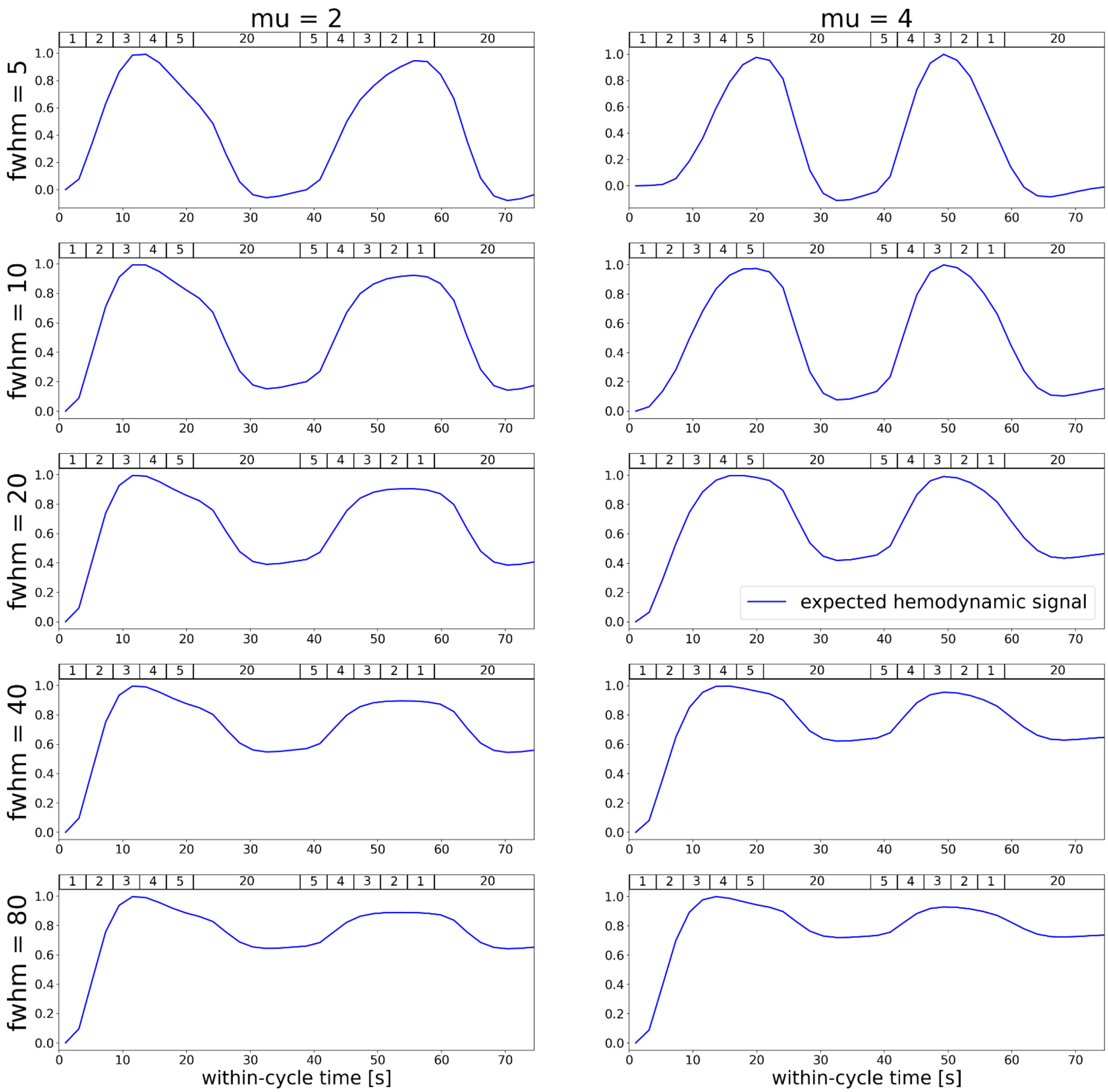

Figure S12: Simulated effect of tuning parameters on numerosity-related time courses. Panels display expected hemodynamic signals when generating neuronal signals given typical tuning parameters (logarithmic tuning function; left: mu = 2; right: mu = 4; from top to bottom: fwhm = 5, 10, 20, 40, 80; cf. Figure 5c,d) and convolving these signals with the canonical hemodynamic response function. These signals show that differential responses to numerosities can emerge even with high tuning width for both, low and medium preferred numerosities.

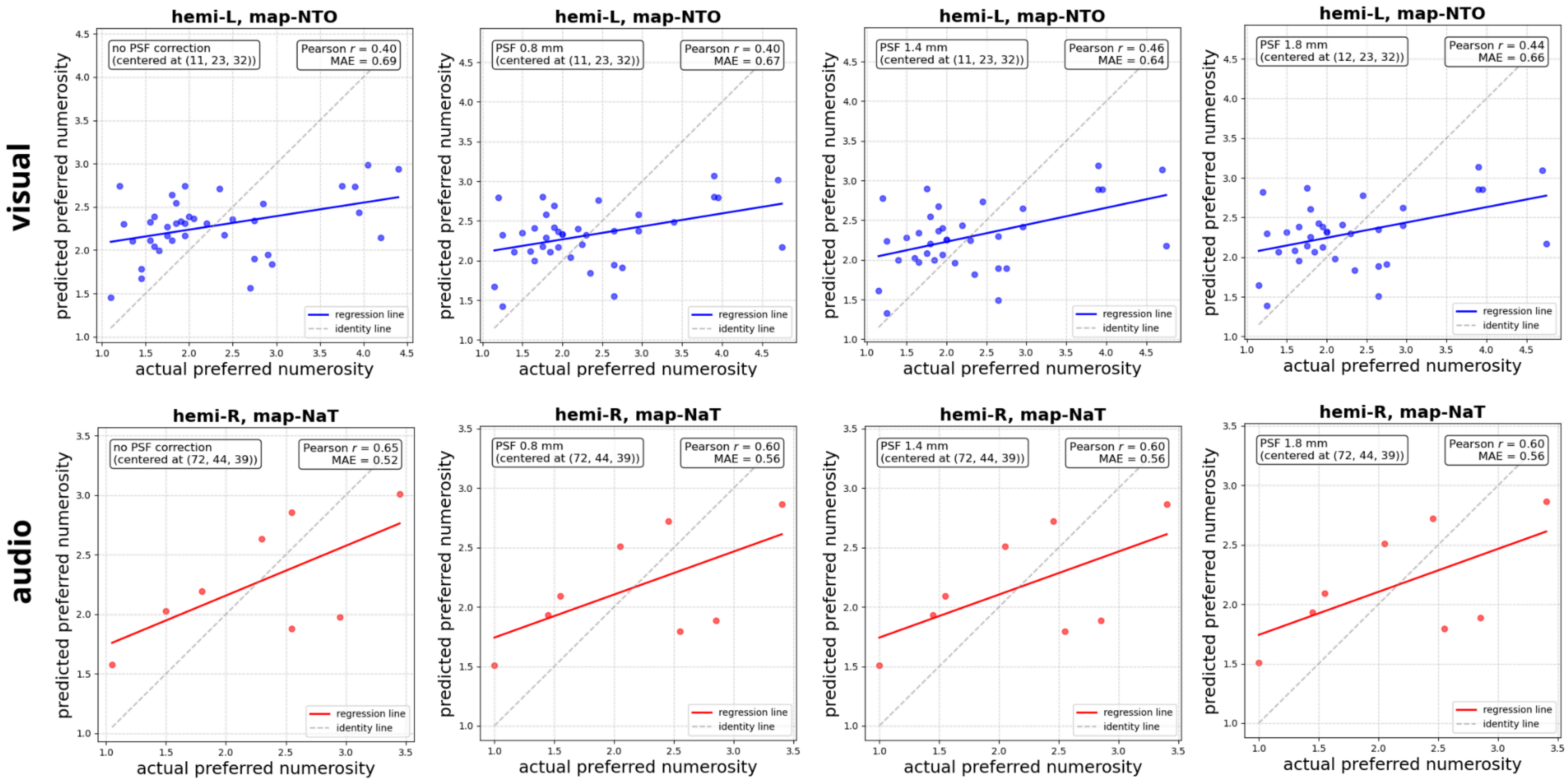

Figure S13: Fitted and preferred numerosity of numerotopic maps based on volumetric analysis. Scatter plots show preferred numerosity predicted from voxel coordinates in native space plotted against the actual preferred numerosity. Visual numerosities are shown for numerotopic map NTO in the left hemisphere (Hemi-L, top) and auditory numerosities are shown for numerotopic map NaT in the right hemisphere (Hemi-R, bottom), from two example subjects (same subjects as in Figs. 2, 3, 5). Due to the smaller number of supra-threshold voxels in volumetric clusters, predicted numerosity was computed using simple linear regression, rather than the fifth-order polynomial regression with 10-fold cross-validation used in the surface-based analysis. Wiener deconvolution with Gaussian filters (FWHM = 0.8, 1.4, 1.8 mm) was applied to correct for the point spread function (PSF). The tested Gaussian filter sizes were chosen based on previously reported PSF measurements in human V2 obtained using 7T GE-EPI sequences (Fracasso et al., 2021). Cluster centers are reported in native voxel space. MAE = mean absolute error.

References

1. Harvey, B. M., Klein, B. P., Petridou, N. & Dumoulin, S. O. Topographic representation of numerosity in the human parietal cortex. *Science* **341**, 1123–1126 (2013).
2. Harvey, B. M., Fracasso, A., Petridou, N., & Dumoulin, S. O. Topographic representations of object size and relationships with numerosity reveal generalized quantity processing in human parietal cortex. *Proc. Natl. Acad. Sci. U. S. A.* **112**, 13525–13530 (2015).
3. Fracasso, A., Dumoulin, S. O., & Petridou, N. Point-spread function of the BOLD response across columns and cortical depth in human extra-striate cortex. *Prog. Neurobiol.* **202**, 102034 (2021).
