# Supplementary material for: Population coding for visual and auditory quantity in human numerotopic maps
Source: Commun Biol. 2026 Feb 27;9:383. doi: 10.1038/s42003-026-09752-2 (PMC12992664; doi:10.1038/s42003-026-09752-2)
Supplement: Supplementary file 2 — Reporting Summary [file 42003_2026_9752_MOESM2_ESM.pdf]

nature portfolio

Corresponding author(s): Michael A. Skeide

Last updated by author(s): Jul 25, 2025

Reporting Summary

Nature Portfolio wishes to improve the reproducibility of the work that we publish. This form provides structure for consistency and transparency in reporting. For further information on Nature Portfolio policies, see our Editorial Policies and the Editorial Policy Checklist.

Statistics

For all statistical analyses, confirm that the following items are present in the figure legend, table legend, main text, or Methods section.

n/a	Confirmed	
☐	☒	The exact sample size (*n*) for each experimental group/condition, given as a discrete number and unit of measurement
☐	☒	A statement on whether measurements were taken from distinct samples or whether the same sample was measured repeatedly
☐	☒	The statistical test(s) used AND whether they are one- or two-sided<br>*Only common tests should be described solely by name; describe more complex techniques in the Methods section.*
☐	☒	A description of all covariates tested
☐	☒	A description of any assumptions or corrections, such as tests of normality and adjustment for multiple comparisons
☐	☒	A full description of the statistical parameters including central tendency (e.g. means) or other basic estimates (e.g. regression coefficient) AND variation (e.g. standard deviation) or associated estimates of uncertainty (e.g. confidence intervals)
☐	☒	For null hypothesis testing, the test statistic (e.g. *F*, *t*, *r*) with confidence intervals, effect sizes, degrees of freedom and *P* value noted<br>*Give P values as exact values whenever suitable.*
☒	☐	For Bayesian analysis, information on the choice of priors and Markov chain Monte Carlo settings
☐	☒	For hierarchical and complex designs, identification of the appropriate level for tests and full reporting of outcomes
☐	☒	Estimates of effect sizes (e.g. Cohen's *d*, Pearson's *r*), indicating how they were calculated

Our web collection on statistics for biologists contains articles on many of the points above.

Software and code

Policy information about availability of computer code

Data collection	Python, PsychoPy
Data analysis	Nipype, fMRIprep, FreeSurfer, AFNI, Python (numpy, scipy, pandas, statsmodels, nibabel, surfplot, matplotlib)

For manuscripts utilizing custom algorithms or software that are central to the research but not yet described in published literature, software must be made available to editors and reviewers. We strongly encourage code deposition in a community repository (e.g. GitHub). See the Nature Portfolio guidelines for submitting code & software for further information.

Data

Policy information about availability of data

All manuscripts must include a data availability statement. This statement should provide the following information, where applicable:

- Accession codes, unique identifiers, or web links for publicly available datasets
- A description of any restrictions on data availability
- For clinical datasets or third party data, please ensure that the statement adheres to our policy

After acceptance, the data for this study will be made available through a public link on our GitHub repository (link provided in the manuscript). For peer review, data are available from the corresponding author on reasonable request.

Human research participants

Policy information about studies involving human research participants and Sex and Gender in Research.

Reporting on sex and gender	Sex was determined based on self-reporting. Sex was balanced across participants in study design, such that six male and six female persons participated in this study. Since the studied phenomenon is assumed to be independent of sex, sex was not included into group-based analyses as a covariate. The sex of each individual and all single-subject results are reported.
Population characteristics	Participants were healthy adults at 20 to 34 years of age.
Recruitment	Participants were recruited via the website of the host institution. Self-selection bias and other biases cannot be excluded. These biases are unlikely to impact the results of the present study which targets very basic sensory-cognitive capacities that were replicated in most individual participants.
Ethics oversight	The study was approved under the reference 317/19-ek by the Ethics Committee at the Medical Faculty of the University of Leipzig, Germany (IRB00001750).

Note that full information on the approval of the study protocol must also be provided in the manuscript.

Field-specific reporting

Please select the one below that is the best fit for your research. If you are not sure, read the appropriate sections before making your selection.

☒ Life sciences ☐ Behavioural & social sciences ☐ Ecological, evolutionary & environmental sciences

For a reference copy of the document with all sections, see nature.com/documents/nr-reporting-summary-flat.pdf

Life sciences study design

All studies must disclose on these points even when the disclosure is negative.

Sample size	No sample-size calculation was performed. Our analysis is based on 24 datasets collected from 12 participants. Given that all 24 datasets were in agreement and lead to similar conclusions with high statistical confidence, we did not consider it as scientifically necessary, economically and ecologically reasonable and ethically plausible to include more participants.
Data exclusions	No subjects were excluded. Within subjects, cortical surface vertices which did not exhibit tuning to numerosity according to our numerosity receptive field model were excluded from further analysis (see Methods).
Replication	The main experimental effects of interest were analyzed within each participant (across 8 runs, each including 4 cycles, each including 6 different numerosity levels in repeated presentations) and across 12 independent participants (see Results).
Randomization	Subjects were randomly drawn from the population of young adults (18-35 yrs). Due to the recruiting process, there may be a bias towards more educated young adults. All subjects were administered the same experimental procedure.
Blinding	Blinding was not relevant to this non-interventional study.

Reporting for specific materials, systems and methods

We require information from authors about some types of materials, experimental systems and methods used in many studies. Here, indicate whether each material, system or method listed is relevant to your study. If you are not sure if a list item applies to your research, read the appropriate section before selecting a response.

Materials & experimental systems

n/a	Involved in the study	
☒	☐	Antibodies
☒	☐	Eukaryotic cell lines
☒	☐	Palaeontology and archaeology
☒	☐	Animals and other organisms
☒	☐	Clinical data
☐	☒	Dual use research of concern

Methods

n/a	Involved in the study	
☒	☐	ChIP-seq
☒	☐	Flow cytometry
☐	☒	MRI-based neuroimaging

Dual use research of concern

Policy information about [dual use research of concern]

Hazards

Could the accidental, deliberate or reckless misuse of agents or technologies generated in the work, or the application of information presented in the manuscript, pose a threat to:

No	Yes	
☒	☐	Public health
☒	☐	National security
☒	☐	Crops and/or livestock
☒	☐	Ecosystems
☒	☐	Any other significant area

Experiments of concern

Does the work involve any of these experiments of concern:

No	Yes	
☒	☐	Demonstrate how to render a vaccine ineffective
☒	☐	Confer resistance to therapeutically useful antibiotics or antiviral agents
☒	☐	Enhance the virulence of a pathogen or render a nonpathogen virulent
☒	☐	Increase transmissibility of a pathogen
☒	☐	Alter the host range of a pathogen
☒	☐	Enable evasion of diagnostic/detection modalities
☒	☐	Enable the weaponization of a biological agent or toxin
☒	☐	Any other potentially harmful combination of experiments and agents

Magnetic resonance imaging

Experimental design

Design type: continuous stimulation, event-related fMRI

Design specifications: Each of the 8 functional MRI runs per subject consisted of 4 cycles which in turn consisted of 6 different (visual or auditory) stimuli. Stimuli were presented for 300 ms with 400 ms break (visual) or for 500 ms with 200 ms silence (auditory). Six repetitions per stimulus resulted in a presentation duration of 4,200 ms per stimulus. There were no inter-stimulus-intervals and no inter-cycle-intervals. Four cycles resulted in a total duration of 302.4 s per run.

Behavioral performance measures: No numerosity judgments were required. To ensure that participants were paying attention to the stimuli, they were instructed to press a button whenever they heard a high-pitch tone sequence (10% of all stimulus events in the auditory experiment) or when they saw white instead of black dot patterns (10% of all stimulus events in the visual experiment). The average hit rate is reported for each modality and participant.

Acquisition

Imaging type(s): functional magnetic resonance imaging (fMRI)

Field strength: 7T

Sequence & imaging parameters: T2*-weighted scans were acquired using a gradient-echo echo-planar imaging (EPI) sequence (sagittal orientation, 41 slices, thickness = 1.75 mm, field of view = 192 mm x 192 mm, matrix size = 110 x 110, voxel size = 1.75 x 1.75 x 1.75 mm, TR = 2,100 ms, TE = 24 ms, flip-angle = 70°).

Area of acquisition: We conducted whole-brain scans only omitting the most anterior parts of the frontal and temporal lobes, where ultra-high-field fMRI at 7 Tesla has low response amplitudes and large spatial distortions.

Diffusion MRI ☐ Used ☒ Not used

Preprocessing

Preprocessing software: Preprocessing was performed using FreeSurfer 7.4.123 and fMRIprep 23.1.424, based on Nipype 1.8.625.

Normalization	Data were not normalized to enable single-subject analyses in native space.
Normalization template	The functional time-series were resampled onto the fsnative (individual participant) and fsaverage (standard space) surfaces following the FreeSurfer reconstruction nomenclature.
Noise and artifact removal	Before estimation of tuning parameters, preprocessed fMRI signals were standardized to units of percent signal change and 12 confound variables were regressed from standardized signals (six motion parameters; WM, CSF and global signal; three cosine regressors). Then, signals were averaged across runs and numerosity analysis was performed for a single averaged run.
Volume censoring	Volume censoring was not applied.

Statistical modeling & inference

Model type and settings	A population receptive field model describing neural and hemodynamic responses to presented numerosity was fitted to each individual vertex in a mass-univariate fashion. Estimated parameters from this model (preferred numerosity, tuning widths, scaling factor) were extracted from this model and submitted to analyses across vertices and subjects.
Effect(s) tested	At the single-subject level, statistical significance of numerosity selectivity was determined by comparing the receptive field model against a null model not accounting for presented numerosity. Extracted parameters were submitted to correlational analyses to identify relationships with cortical geometry. Linear mixed-effects models with subject as random effect were estimated to test effects of preferred numerosity on tuning width and cortical surface area at the group level.

Specify type of analysis: ☐ Whole brain ☐ ROI-based ☒ Both

Anatomical location(s)	Regions of interest were obtained as those clusters of numerosity-selective vertices that were close to previously reported numerosity-selective maps in standard space and exceeded a specific cortical surface threshold (visual: 50 mm², auditory: 25 mm²).
Statistic type for inference (See Eklund et al. 2016)	All clusters were analyzed separately, such that no cluster correction was applied.
Correction	The voxel-wise threshold ($R^2 = 0.2$) corresponded to an uncorrected p-value of p = 6.4 · 10^{-7}.

Models & analysis

n/a	Involved in the study
☒	☐ Functional and/or effective connectivity
☒	☐ Graph analysis
☐	☒ Multivariate modeling or predictive analysis

Multivariate modeling and predictive analysis	Multiple linear regression was used to predict preferred numerosity (dependent variable) of numerosity-selective clusters based on pial x-, y- and z-coordinates in standard space (independent variables). Correlation coefficient was used as measure of predictive performance.
